# Antibiotic resistance, virulence gene, phylogenetic group and genetic diversity of *Escherichia coli* isolated from Tibetan pig farms in Garze Tibetan Autonomous Prefecture, Sichuan, China

**DOI:** 10.3389/fcimb.2025.1526028

**Published:** 2025-05-12

**Authors:** Xing Zhao, Jun Liang, Zhaobin Xia, Chaoxi Chen

**Affiliations:** ^1^ College of Animal and Veterinary Sciences, Southwest Minzu University, Chengdu, China; ^2^ Institute of Veterinary Drug and Feed Product Safety Testing, Zhengzhou Inspection and Testing Center of Product Quality, Zhengzhou, China

**Keywords:** Tibetan pigs, diarrheagenic *Escherichia coli*, antibiotic resistance genes, integrase genes, virulence genes

## Abstract

**Introduction:**

To investigate the correlations among antibiotic resistance, virulence gene, phylogenetic group, and genetic diversity, providing essential data for *Escherichia coli (E. coli)* infection prevention and control in Tibetan pigs.

**Methods:**

A total of 244 *E. coli* isolates were collected. Antimicrobial susceptibility was assessed using the microdilution method. PCR was used to detect antibiotic resistance genes (ARGs), virulence genes, and phylogenetic groups. Genetic diversity was analyzed using enterobacterial repetitive element sequence-based PCR. Enteroaggregative *E. coli* (EAEC) 5-12, a representative strain with multidrug resistance and strong biofilm-forming ability, harboring abundant virulence genes, was selected for whole-genome sequencing (WGS) to validate PCR results.

**Results:**

Among the 244 isolates, 84.43% showed multidrug resistance (MDR), with the highest resistance rates for chloramphenicol (99.59%), sulfadiazine (96.31%), and sulfamethoxazole (93.85%). Twenty-five ARGs were detected, with *ant(3’)-Ia*, *bla*_TEM_, *aac(3’)-II*, *floR*, and *qnrS* exceeding 80% detection rates. Integrase genes intl1 and intl2 were found in 90.16% and 15.16% of isolates, respectively. Seventeen virulence genes were detected; *bcsA* (98.77%), *fimC* (89.75%), and *agn43* (59.43%) were the most prevalent. A total of 106 virulence patterns were identified, with *agn43/bcsA/fimC* being predominant (17.92%). Most strains belonged to phylogenetic group A (45.90%), followed by B1 (34.43%), while 29 strains were unclassified. Sixty-four isolates were identified as diarrheagenic *E. coli* (DEC), predominantly enteroaggregative *E. coli* (EAEC, 90.63%). Biofilm-forming ability was categorized as strong (4.69%), moderate (21.88%), weak (59.38%), or absent (14.06%). Clustering based on 61.2% similarity grouped the 64 DEC into five clusters, with 84.38% in cluster II, which contained all strong biofilm producers.

**Discussion:**

Antimicrobial resistance profiles of EAEC 5–12 confirmed that primarily confer resistance through antibiotic efflux, target alteration, and reduced permeability. These findings will contribute to further understanding the positive correlation between antibiotic resistance and pathogenicity in *E. coli* from Tibetan pig farms, shedding light on the rational use of antimicrobial agents and tackling the antibiotic resistance crisis in Tibetan pig breeding in Garze Tibetan Autonomous Prefecture, Sichuan, China.

## 1 Introduction

*Escherichia coli* (*E. coli*), a facultative anaerobe, is widely present in the gastrointestinal tracts of warm-blooded animals. When the immune response of the host is impaired, certain serotypes of *E. coli* can invade deeper tissues or organs, causing diseases such as diarrhea, pneumonia, and septicemia to acquire antibiotic resistance genes (ARGs) and virulence genes, adapt to environmental changes, and lead to gut microbiota disturbance (Geurtsen et al., 2022). Nearly 70% of antibiotics globally are used in animal production, with this widespread usage contributing significantly to bacterial resistance in pathogens like *E. coli* due to selective pressure and environment acquisition of resistance genes (Gasparrini et al., 2020; McKernan et al., 2021). China is the largest producer and user of antibiotics, with a large share applied in animal production and agriculture (Zhang et al., 2015). In 2023, *E. coli* isolated from Tibetan pigs in Nyingchi, Tibet, China, demonstrated resistance to multiple antibiotics (Cao et al., 2023). Similar resistance was observed in *E. coli* isolated from pigs, yak, beef and dairy cattle, reducing the efficacy of diminished the efficacy of *β*-lactams, quinolones, and aminoglycosides (Kylla et al., 2020; Wang et al., 2021).

Bacteria acquire resistance genes is a significant mechanism for the spread of resistance among bacteria. Integrons, a mobile genetic element, capture and integrate foreign genes, particularly resistance genes, through integrase enzymes (Hall and Collis, 1995).

*E. coli* acquires specific virulence factors through horizontal gene transfer and adaptive evolution, which enhances its adaptability and enables it to infect the host (Kaper et al., 2004; Arnold et al., 2022). Its pathogenicity is linked to virulence factors, typically encoded by genes on chromosomes, plasmids, or other genetic elements. Previous studies have shown that these factors are linked to gastrointestinal diseases (Singh et al., 2019).

*E. coli* is classified into phylogenetic groups based on the presence/absence of genes like *chuA*, *yjaA*, *TspE4.C2*, *arpA*, and *trpA*. Experimental results show differences in pathogenicity, phenotype, and genotype among strains from different phylogenetic groups (Tenaillon et al., 2010). Compared to other phylogenetic groups, *E. coli* in group B2 exhibits a longer survival time in the host’s intestine, which is suspected to be related to the virulence genes carried (Johnson and Kuskowski, 2000; Johnson and Stell, 2000; Escobar-Páramo et al., 2004). Potential pathogenic strains were also found in group D, while strains in groups A and B are mostly commensal *E. coli* (Picard et al., 1999; Duriez et al., 2001).

Tibetan pigs, physiologically adapted to high altitudes, are food animals vulnerable to outbreaks of fatal diarrhea caused by pathogenic *E. coli*, raising serious concerns for both animal and human health. Understanding the virulence genes, phylogroups, and phenotypic resistance characteristics in *E. coli* strains in Tibetan pigs is essential.

The linkages between virulence determinants and antibiotic resistance are still not clear despite several studies. It is important to assess the risk of antibiotic resistance and virulence factors on public health and this necessitates additional studies on such neglected food animals. This study aimed to investigate antibiotic resistance, virulence genes, and phylogenetic group distribution of *E. coli* isolated from Tibetan pig farms in Garze Tibetan Autonomous Prefecture, Sichuan, China. Findings provide foundational data on the relationship between antibiotic resistance and pathogenicity in *E. coli* from Tibetan pigs, supporting the rational use of antimicrobials and helping curb the spread of bacterial resistance in the Tibetan pig industry.

## 2 Materials and methods

### 2.1 Samples collection and reference strain

From 19 June to 6 October 2022, 301 samples (247 feces, 41 soil, and 23 water) were aseptically collected from Tibetan pigs in Garze Tibetan Autonomous Prefecture, Sichuan Province (**Supplementary Table S1**). Seven farms were sampled (Luding, 4 farms; Xiangcheng, 1 farm; and Daocheng, 4 farms) and samples were transported to Southwest Minzu University in ice-cooled containers for *E. coli* isolation and identification within 72 h. Anal swabs from normal animals, surface soil, and drain water were collected from intensive farms using sterile plastic bags. The reference strain ATCC25922 was kept in the Laboratory of Veterinary Pharmacology and Toxicology of Southwest Minzu University.

### 2.2 Antibacterial agents, medium and molecular biology reagents

Twenty-three antibacterial agents including Tetracyclines (Doxycycline, Oxytetracycline, Tetracycline), Sulfonamides (Sulfamethoxazole, Sulfadiazine), *β*-lactams (Amoxicillin/clavulanic acid, Ampicillin, Cefoxitin, Cefquinome, Oxacillin), Amphenicols (Chloramphenicol, Florfenicol), Rifamycins (Rifampin), Aminoglycosides (Gentamicin, Kanamycin, Spectinomycin, Streptomycin), Fluoroquinolones (Ciprofloxacin, Enrofloxacin, Nalidixic acid, Sarafloxacin), Polypeptides (Polymyxin B), Polyphosphates (Fosfomycin) were purchased from Shanghai YuanYe Biotechnology Co., Ltd. Tryptone soy broth (TSB), MacConkey agar (MAC), *E. coli* coliform chromogenic agar, Eosin methylene blue agar (EMB), Mueller-Hinton broth (MH), and Gram staining kits were provided by Qingdao Haibo Biotechnology Co., Ltd. molecular biology reagents for PCR including DL2000 DNA marker and 2×*Taq* Master Mix were purchased from Vazyme Biotech Co., Ltd (Nanjing, China).

### 2.3 Isolation, identification, and DNA extraction of *E. coli*

A suitable volume of environmental samples (0.2 g feces, 0.5 g soil, and 5 mL water) was placed into a test tube containing 5 mL of TSB and incubated for 12 h at 37°C. A loop of the bacterial suspension was streaked on MAC agar and incubated at 37°C for 12 h. Single colonies of moderate size with pink color were sequentially inoculated to EMB, *E. coli*/coliform color indicator medium, and MAC. The purified single colonies were subjected to the suspected *E. coli* via Gram staining and microscopic examination. PCR reaction targeting the *β*-glucosidase gene *uidA* (*uidA*-F: ATGCCAGTCCAGCGTTTTTGC and *uidA*-R: AAAGTGTGGGTCAATAATCAGGAAGTG) was amplified and the PCR products were subjected to the NCBI BLAST platform for sequence analysis.

DNA was extracted using the nuclease-free water boiling method and used as the DNA template in all the following molecular biological tests.

### 2.4 Antimicrobial sensitivity testing

The broth microdilution method used for the antimicrobial sensitivity profile and the obtained MICs (minimum inhibitory concentration) were interpreted according to the Clinical and Laboratory Standards Institute (CLSI) guidelines (CLSI, 2013). The reference strain ATCC25922 was used as a quality control. Briefly, dispense 100 µL of MH medium into each well in the columns from 1 to 12 and 100 µL in column 1 using a 300 µL electronic multichannel pipette (Eppendorf, Germany). Then, 100 µL of appropriate 4-fold concentrated stock solutions (5012 μg/mL) was pipetted into the wells in column 1 (antibiotic concentration will be diluted this way 1:1) and mixed, 100 µL of the mixed solutions in column 1 was withdrawn and moved into the column 2. Repeat the dilution procedure up to column 10 and additionally discard 100 µl of solutions from this last column. 100 µL of the suspension (10^6^ CFU/mL) were inoculated in columns 1 to 10, columns 11 and 12 having a positive (no antibiotic) and negative growth control for medium sterility. Finally, transfer microtiter plates into the closable tray until inoculation is 12–16 h. Multi-drug resistance (MDR) was determined according to the reference (Magiorakos et al., 2021).


Concordance rate=

$$\frac{\text{Number of strains with resistance genes and corresponding resistance phenotypes}}{\text{Number of strains with resistance phenotypes}}x100\%$$

Consistency rate=

$$\frac{\text{Number of strains with resistance genes and corresponding integrase genes}}{\text{Number of strains positive for integrase genes}}x100\%$$

### 2.5 Detection of ARGs, integrase genes, and virulence genes

Specific primers for antibacterial resistance, integrase, and virulence genes were designed according to the literature (**Supplementary Table 2**) and synthesized by Sangon Biotech (Shanghai) Co., Ltd. Primers for the detection of ARGs and integrase genes were chosen based on the antibiotic categorizations. Virulence genes were chosen based on their functional characteristics. Singleplex PCR amplification reactions were carried out in 25 µL volumes comprising 2 µL genomic DNA, 12.5 µL 2×*Taq* Master Mix, 1 µL of each primer, and 8.5 µL ddH_2_O. The thermocycler conditions were as follows: denaturation at 95°C for 5 min followed by 35 cycles of denaturation at 95 °C for 30 s, variable annealing for 30 s, and extension at 72 °C for 15 s (**Supplementary Tables S2** and **S3**). Finally, a final extension for 10 min at 72°C. The PCR products were analyzed using by 1% agarose gel electrophoresis staining with ethidium bromide. Concordance rates between drug-resistance phenotypes and genotypes were calculated, and the consistency rates of ARGs and integrase genes were assessed (An, 2024). Chi-Plot was used to investigate the possible association of two variables (https://www.chiplot.online/).

### 2.6 Phylogenetic analysis

Phylogenetic analysis of the isolated *E. coli* using a set of genes *chuA*, *yjaA*, *TspE4.C2*, *arpA*, *arpAgpE*, *trpAgpC*, *trpBA* were detected by multiplex PCR assays and the results were interpreted as previously described (Olivier et al., 2013). Primer information is listed in **Supplementary Table S4**. Quadruplex genotypes and steps for assigning *E. coli* isolates to phylogroups are listed in **Supplementary Table S5**.

### 2.7 Determination, genetic diversity, and biofilm formation of diarrheagenic *E. coli* (DEC)

DEC was determined according to the National Food Safety Standard GB/T 4789.6-2016 (National Health and Family Planning Commission of the People’s Republic of China, 2016). The criteria for diarrheagenic *E. coli* genetic typing are listed in **Supplementary Table S6**. ERIC-PCR typing was conducted to assess the genetic diversity of DEC by amplifying repetitive DNA sequences found in the intergenic regions and the primers information was shown in **Supplementary Table S4**.

ERIC-PCR reactions were performed in 25 μL volumes containing 1 μL of each primer, 12.5 μL of the master mix (CinnaGen, Iran), 2 μL of DNA template, and 8.5 μL of deionized water. The ERIC-PCR reaction program was performed following initial denaturation at 95°C for 5 min, with the next 35 cycles consisting of a denaturation step at 95°C for 1 min, annealing at 53°C for 1 min, extension at 72°C for 4 min, and a final extension for 10 min at 72°C. 5 μL of ERIC-PCR products were loaded on 1% agarose gel and conducted at 120 V for 30 min. Meanwhile, a 100-base pair-DNA marker was used as a standard measuring means. DNA bands were visualized with a ChemiDoc MP Imaging System (BIO-RAD).

ERIC-PCR bands were used to construct a dendrogram by converting into a binary matrix where each band is either present (1) or absent (0) for each isolate. The dendrogram for the 64 strains was constructed in NTSYS software using the Unweighted Pair Group Method with the Arithmetic mean (UPGMA) method.

The biofilm-forming ability of DEC was semi-quantitative determined using crystal violet assay (Chen et al., 2021), and the optical density threshold value (OD_C_) was set as the average value of the negative control plus three standard deviations. The biofilm formation ability was categorized as follows: OD_570nm_ ≤ OD_C_ indicates no biofilm formation, OD_C_ < OD_570nm_ ≤ 2 OD_C_ indicates weak biofilm formation, 2 OD_C_ < OD_570nm_ ≤ 4OD_C_ indicates moderate biofilm formation, and D_570nm_ > 4 D_C_ indicates strong biofilm formation.

### 2.8 Whole genome sequencing of EAEC 5-12

To confirm the presence of resistance-related genes and allelic variations in EAEC 5-12(multidrug resistance and strong biofilm-forming ability, harboring abundant virulence genes), the amino acid sequence from WGS data was analyzed using CARD’s Resistance Gene Identifier (RGI) software. A single colony was selected for enrichment in a 5 mL aliquot of TSB, and bacterial culture at the logarithmic phase was centrifuged at 4000 g for 10 min at 4°C. The supernatant was discarded, and the pellet was washed twice with sterile water. Samples were shipped with dry ice for Illumina sequencing (Beijing Novogene Bioinformatics Technology Co., Ltd). After sequencing and gene function prediction, the antimicrobial resistance genes were verified against the NCBI AMRFinderPlus database. For virulence genes, DIAMOND software was used to compare the non-redundant gene set with the VFDB core database, applying a cut-off of 80% identity and 70% coverage (parameters: BLASTP; E-value ≤ 1e-5).

### 2.9 Statistics and data analysis

Statistical analysis was performed using WHONET 5.6 and IBM SPSS Statistics 29. The chi-squared test and *Fisher’s* exact test were used to analyze differences, with *P*< 0.05 considered statistically significant.

## 3 Results

### 3.1 Isolation and identification of *E. coli*

**Figure 1:**
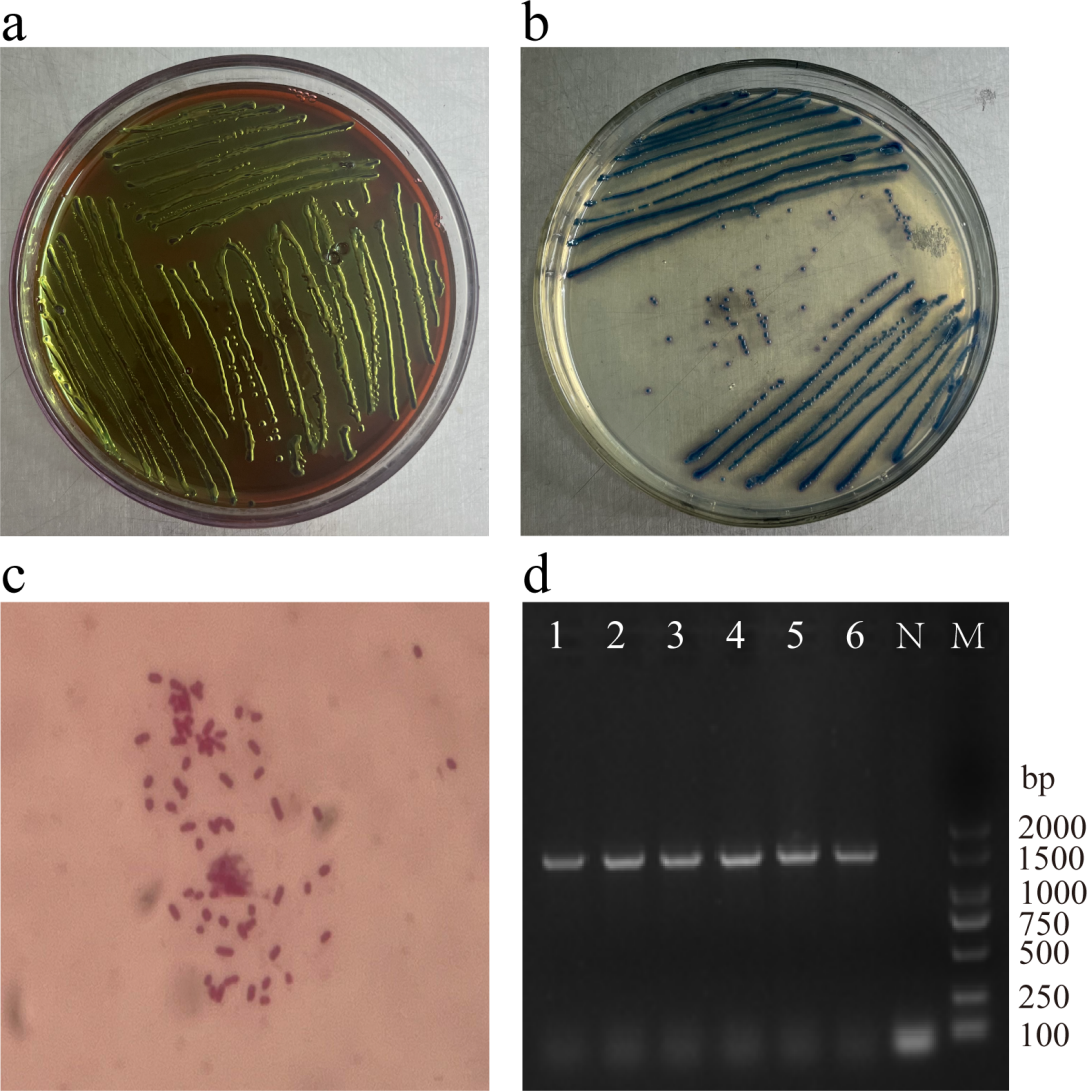
Identification of *E. coli* by combining morphological and molecular biological methods. **(a)** Colonies showing greenish metallic sheen on EMB agar; **(b)** Dark blue to violet colonies on *E. coli* coliform chromogenic agar; **(c)** *E coli* Colony Gram staining (1000×); **(d)** *E. coli* marker gene *uidA* (Lane 1-6: Representative strains. N, Negative control; M, DL 2000 DNA Marker).

Species identification was conducted using a combination of morphological and molecular biological methods (**Figures 1a-c**). The *E. coli* marker gene *uidA* was detected in 81.06% (244/301) of strains, with a molecular weight of approximately 1487 bp (**Figure 1d**). Of these, 64 strains (26.23%, 64/244) were identified as DEC. Among the isolates, the percentage of *E. coli* isolated from feces, soil, and water was 72.09% (217/301), 2.33% (7/301), and 6.64% (20/301), respectively.

### 3.2 Antimicrobial resistance and multi-drug resistance analysis

**Figure 2:**
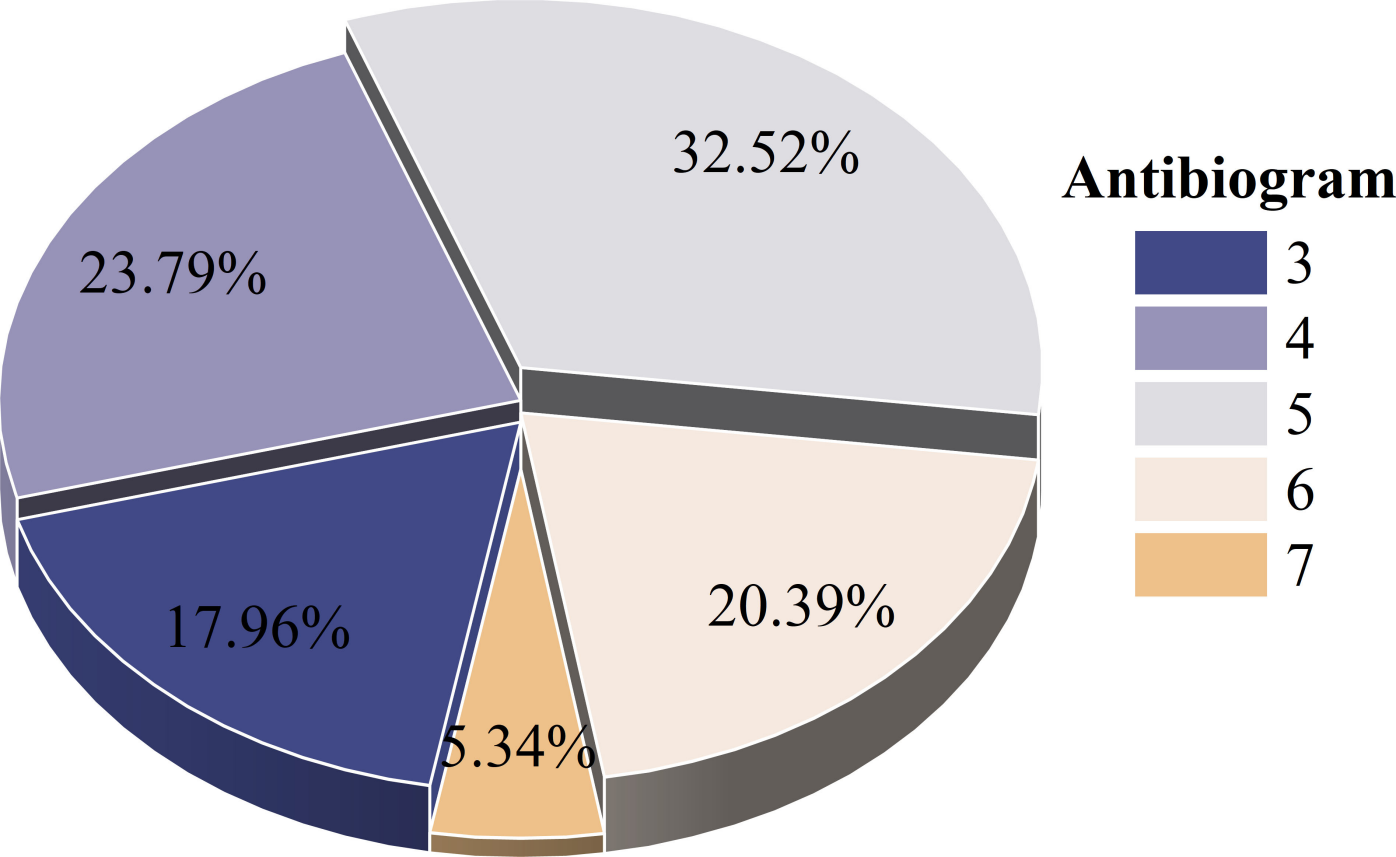
Antibiogram of MDR *E. coli*. (3–7 means that resistance to 3–7 kinds of antibiotics was tested).

The antimicrobial sensitivity testing of 244 *E. coli* strains against 23 antibiotics is summarized in **Table 1**. The strains exhibited resistance to various antibiotics, including aminoglycosides, sulfonamides, and tetracyclines, with resistance rates exceeding 50%. The highest resistance rates were observed for CHL, SMZ, and SMX, posing 99.59%, 96.31%, and 93.85%, respectively. However, the isolated strains were sensitive to PLB (93.85%), CEF (91.8%), TIO (88.52%), CIP (88.11%), GEN (87.3%), KAM (85%). 84.43% (206/244) of the *E. coli* strains displayed MDR, with the highest proportion (32.52%, 67/206) being resistant to five classes of antibiotics (**Figure 2**).

**Table 1:** Antibiotic sensitivity testing of 244 *E. coli* isolated from Tibetan pigs.

Antibiotic classes	Names of antibiotics	R (%)	I (%)	S (%)	MIC_50_	MIC_90_	MIC range
Tetracyclines	OTC	69.67	10.25	20.08	64	256	0.5 - 256
	DOX	51.64	9.84	38.52	8	32	0.25 - 128
	TCY	64.34	2.05	33.61	64	256	0.25 - 256
Sulfonamides	SMZ	96.31	0.00	3.69	512	512	32 - 512
	SMX	93.85	0.00	6.15	512	512	8 - 512
*β*-lactams	AMP	59.84	1.23	38.93	256	256	2 - 256
	OXA	62.70	29.10	8.20	32	256	2 - 256
	AMC	6.56	22.54	70.90	8	16	0.5 - 64
	TIO	10.25	1.23	88.52	0.25	8	0.25 - 256
	CEF	7.38	0.82	91.80	0.25	1	0.25 - 256
Amphenicols	FLR	79.10	13.11	7.79	32	256	0.5 - 256
	CHL	99.59	0.00	0.41	256	256	2 - 256
Rifamycins	RIF	8.61	38.52	52.87	4	8	1 - 64
Aminoglycosides	STR	26.64	0.00	73.36	16	256	0.5 - 256
	GEN	12.30	0.41	87.30	0.5	16	0.25 - 256
	SPT	24.59	13.11	62.30	32	256	1 - 256
	KAM	14.75	0.00	85.25	4	256	0.5 - 256
Fluoroquinolones	NAC	21.31	0.00	78.69	4	256	0.25 - 256
	CIP	10.25	1.64	88.11	0.25	1	0.25 - 16
	ENR	14.75	3.69	81.56	0.25	8	0.25 - 128
	SAR	8.61	11.48	79.92	0.25	1	0.25 - 16
Polypeptides	PLB	6.15	0.00	93.85	1	2	0.25 - 256
Polyphosphates	FOS	2.87	4.10	93.03	4	64	0.25 - 256

AMC, Amoxicillin/clavulanic acid; AMP, Ampicillin; CEF, Cefquinome; CHL, Chloramphenicol; CIP, Ciprofloxacin; DOX, Doxycycline; ENR, Enrofloxacin; FLR, Florfenicol; FOS, Fosfomycin; GEN, Gentamicin; KAN, Kanamycin; NAL, Nalidixic acid; OTC, Oxytetracycline; OXA, Oxacillin; PLB, Polymyxin B; RIF, Rifampin; SAR, Sarafloxacin; SMX, Sulfamethoxazole; SMZ, Sulfadiazine; SPT, Spectinomycin; STR, Streptomycin; TCY, Tetracycline; and TIO, Cefoxitin. The MIC_50_, MIC_90_ and MIC range units are μg/mL.

**Figure 3:**
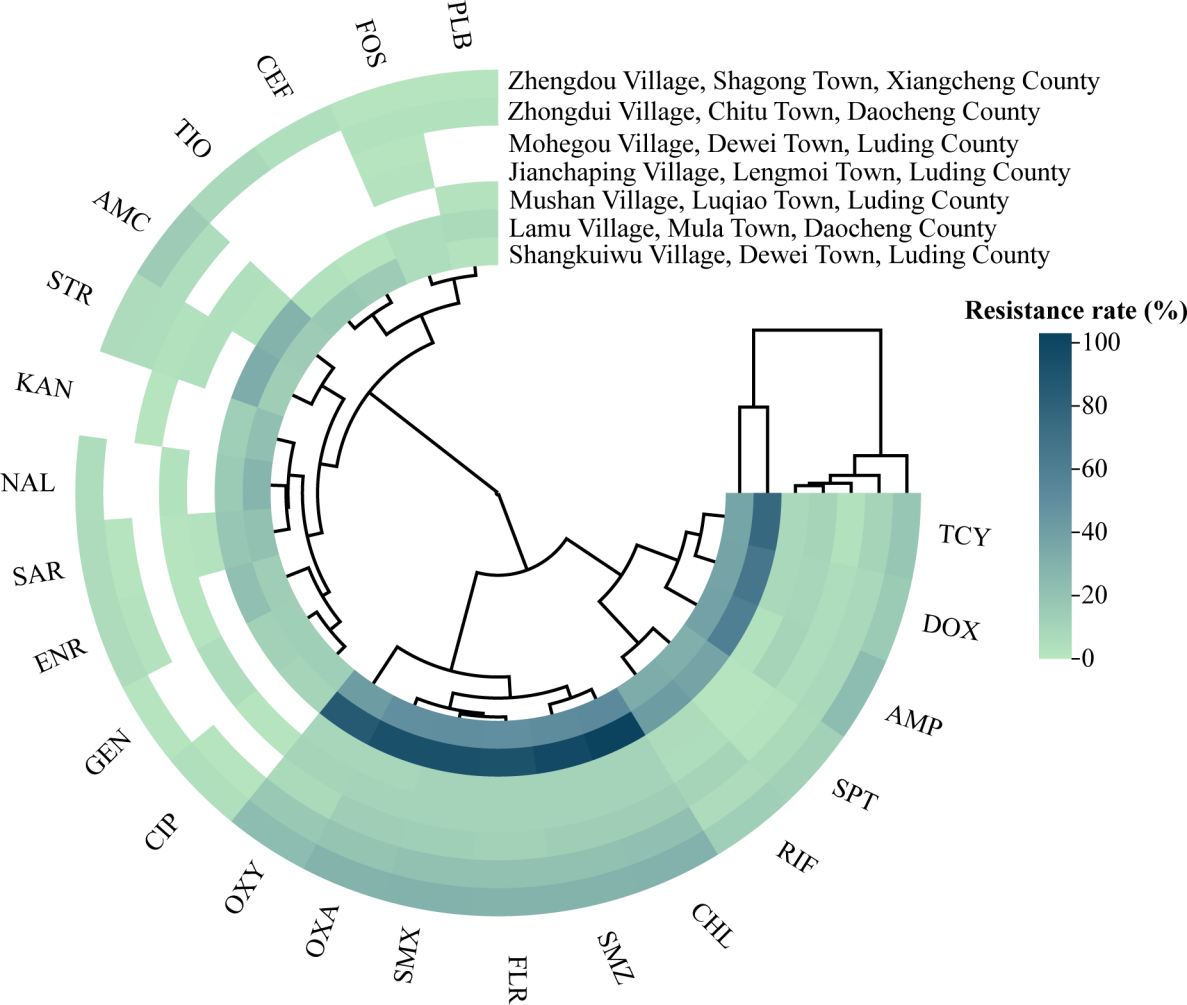
Antibacterial resistance in seven different sampling sites.

Among the seven sampling sites, strains from Shangkuiwu Village and Lamu Village exhibited higher resistance rates to aminoglycosides and sulfonamides, followed by tetracyclines and some *β*-lactams. Resistant strains of all 23 antibiotics were detected in these villages. The resistance profiles of the other five sites were similar (**Figure 3**). Regional differences in the detection rates of antimicrobial agents were observed, with statistical significance (*P<* 0.05) for agents such as Ciprofloxacin, Gentamicin, and Sarafloxacin (**Supplementary Table S7**).

### 3.3 Detection of ARGs, integrase genes, and virulence genes

**Figure 4:**
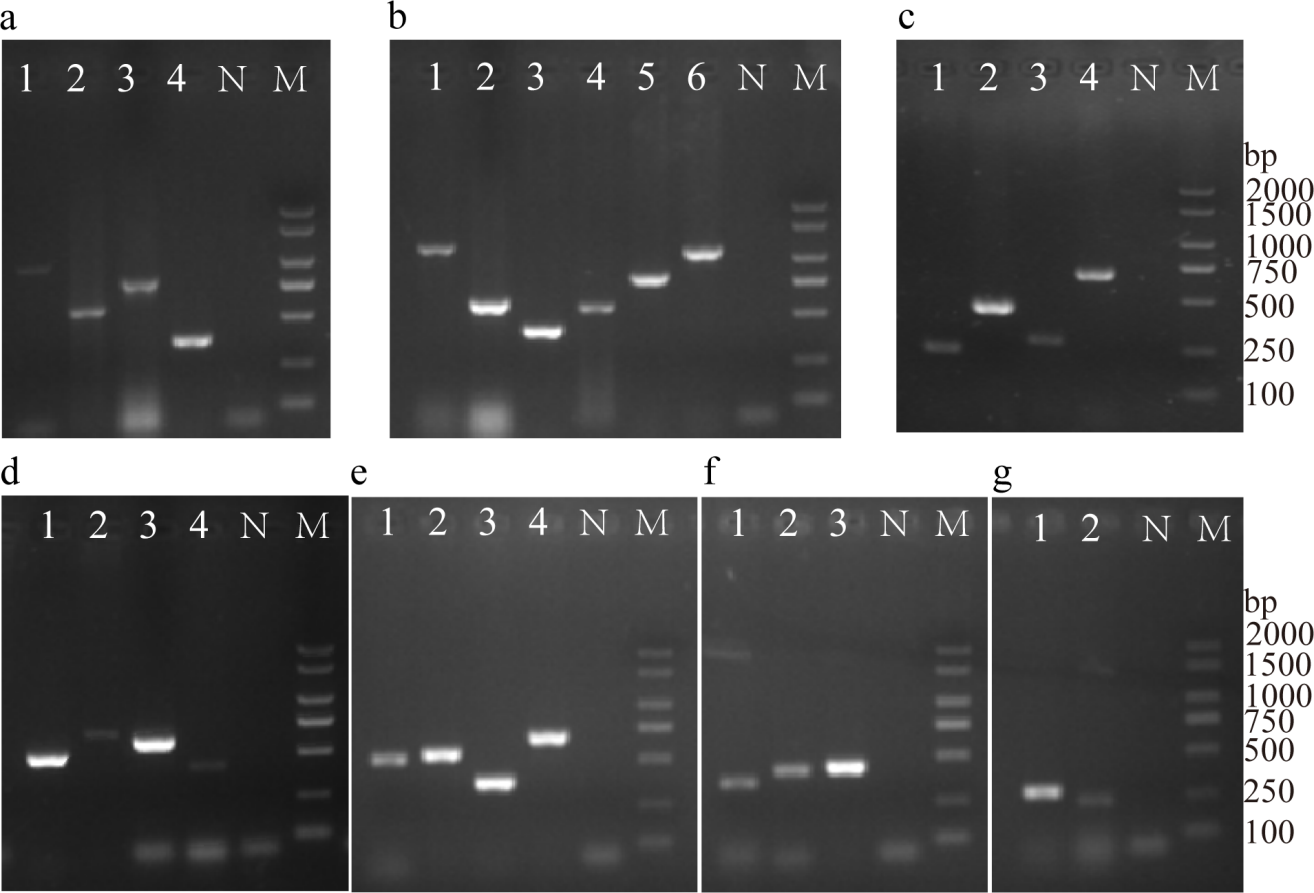
Electrophoresis of PCR amplification products of antibacterial resistance genes and integrase genes. **(a)** Tetracyclines (1-4, *tetA, tetB, tetD, tetM*); **(b)** *β*-lactams (1-6, *blaCMY-2, blaCTX-M-U, blaDHA, blaOXA, blaSHV, blaTEM*); **(c)** Aminoglycosides (1-4, *aac(3’)-II, aadA2, ant(3’)-Ia, aph(3’)-VII*); **(d)** Quinolones (1-4, *aac(6’)-Ib, qnrA, qnrD, qnrS*).; **(e)** Amphenicols (1-4, *cat1, cat2, cmlA, floR*); **(f)** Sulfonamides (1-3, *sul1, sul2, sul3*); **(g)** Integrase (1-2, *intl1, intl2*). (N, Negative control; M, DL 2000 DNA Marker).

**Figure 5:**
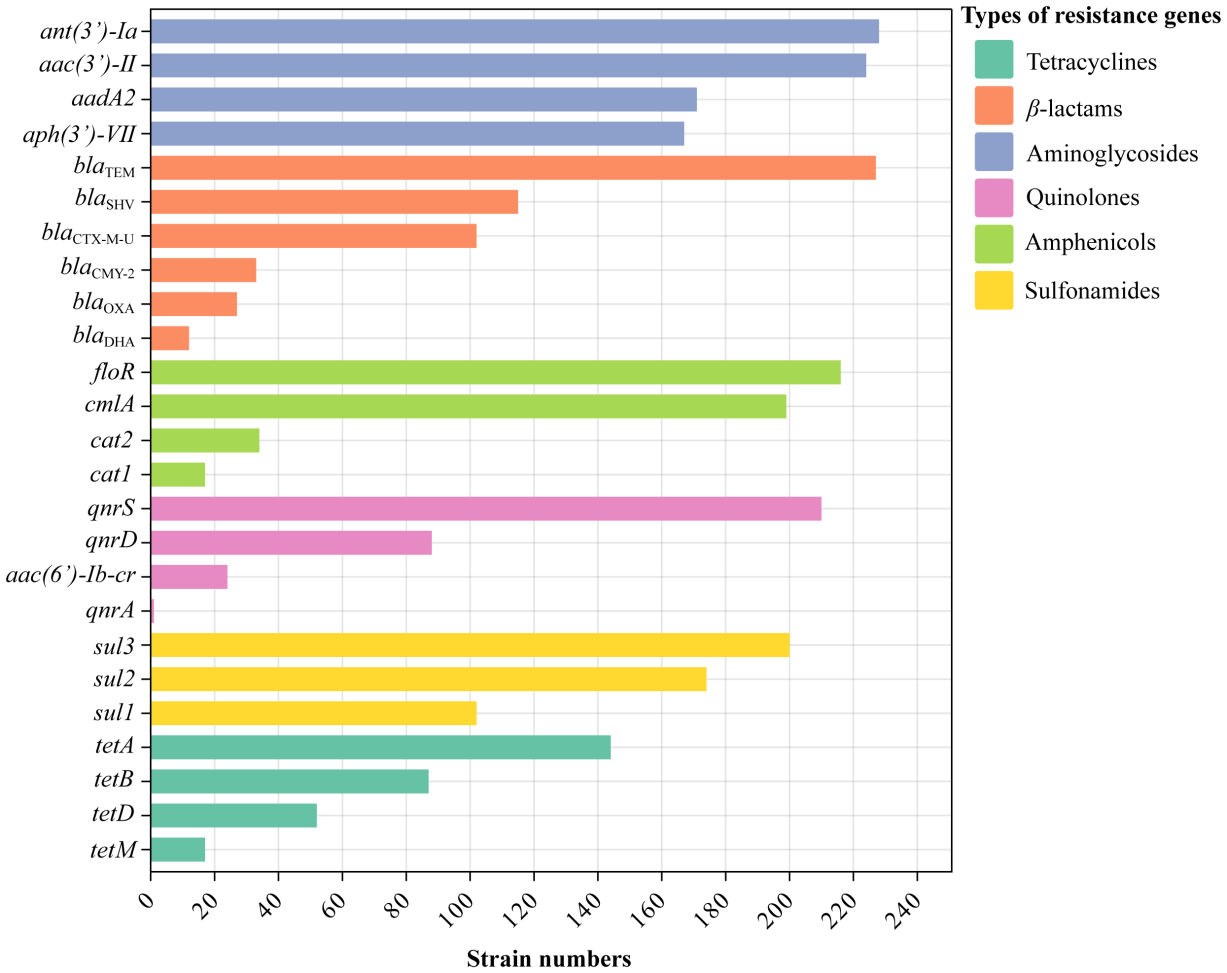
The number of strains with antibacterial resistance genes.

Twenty-five ARGs were positively detected (**Figures 4**, **5**). The predicted fragment length (bp) and optimized annealing temperature (°C) are listed in **Supplementary Table S2**. For ARGs, detection rates exceeded 80% for *ant (3’)-Ia* (93.44%, 228/244), *bla*_TEM_ (93.03%, 227/244), *aac (3’)-II* (91.80%, 224/244), *floR* (88.52%, 216/244), *qnrS* (86.07%, 210/244), *sul3* (81.97%, 200/244), and *cmlA* (81.56%, 199/244). Correlation analysis between ARGs and phenotypes indicated a significant positive association between strains carrying ARGs and those without (**Supplementary Table S8**).

The detection rates of *intl1* and *intl2* were 90.16% (220/244), and 15.16% (37/244), respectively. 14.34% (35/244) of the strains carried both *intl1* and *intl2* genes, and 9.02% (22/244) did not carry the *intl1* and *intl2* genes. Correlation analysis between ARGs and integrase genes was evaluated and shown in **Supplementary Table S9**. Overall, the consistency rate of the integrase gene *intl1* was higher than that of *intl2*, and the detection rate of ARGs in strains carrying integrase genes differed significantly from the strains not carrying integrase genes.

**Figure 6:**
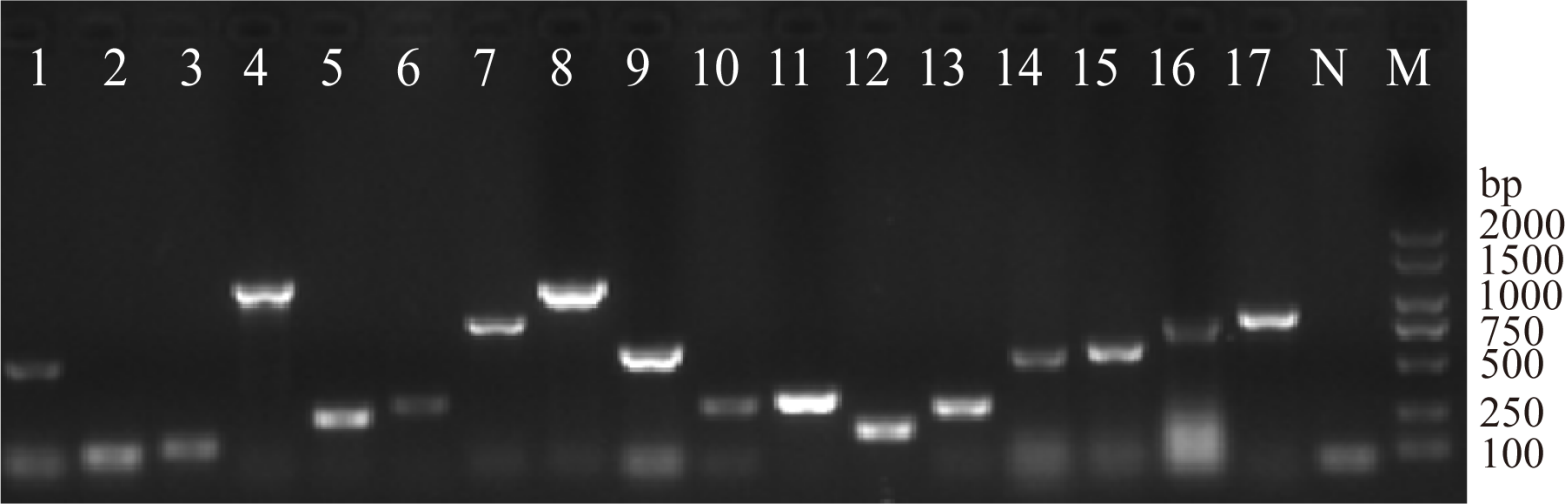
Electropherogram of the PCR amplification product of virulence genes. Lane 1, *agn43*; Lane 2, *astA*; Lane 3, *bcsA*; Lane 4, *colV*; Lane 5, *eaeA*; Lane 6, *fimC*; Lane 7, *fyuA*; Lane 8, *hlyA*; Lane 9, *hlyF*; Lane 10, *irp2*; Lane 11, *iss*; Lane 12, *ler*; Lane 13, *LT*; Lane 14, *ompT*; Lane 15, *sitA*; Lane 16, *stx2*; Lane 17, *tsh*; (N, Negative control; M, DL 2000 DNA Marker).

Among the 17 positively detected virulence genes (**Figure 6**), the highest detection rates for *bcsA*, *fimC*, and *agn43* genes were 98.77% (241/244), 89.75% (219/244), 59.43% (145/244), respectively. However, none of the isolates were positive for *afa*, *bcsB*, *exhA*, *papC*, *stx1* and *vat*. The 244 *E. coli* posed106 virulence genotypes, exhibited the highest number of 11 virulence genes (*agn43*, *astA*, *bcsA*, *colV*, *eaeA*, *fimC*, *hlyF*, *iss*, *ompT*, *sitA*, *tsh*) and the lowest number of 1 virulence gene (*bcsA*). The predominant virulence pattern was *agn43*/*bcsA*/*fimC* (17.92%, 19/106) summarized in **Supplementary Table S10**.

### 3.4 Phylogenetic analysis

**Figure 7:**
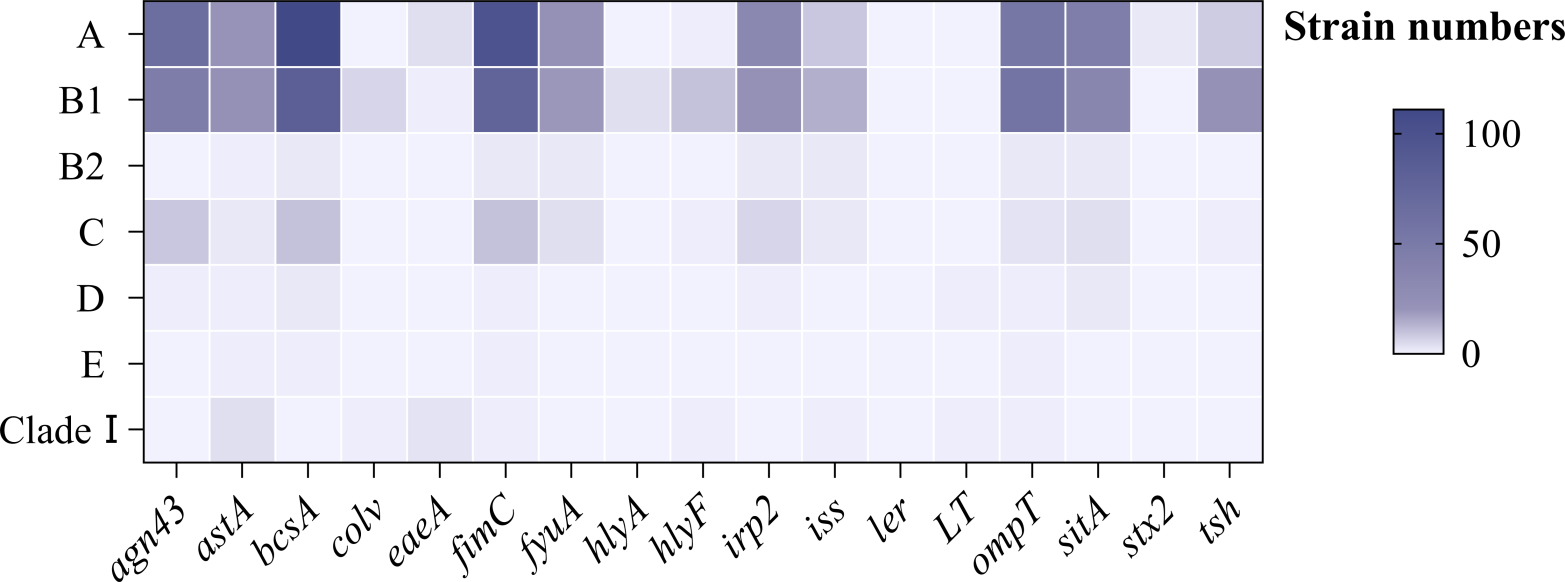
Distribution of virulence genes among phylogenetic groups.

Of the 244 strains, 45.90% (112/244) mainly belonged to group A, followed by group B1(34.43%), group B2(0.82%), group C (4.10%), group D (0.82%), group E (0.41%), and Clade I (1.64%). However, 29 strains could not be classified into any phylogenetic clusters (**Supplementary Figure S1**). **Figure 7** revealed the distribution of virulence genes in 244 *E. coli* among the different phylogenetic groups, the most prevalent genes in group A were *agn43*, *bcsA*, *eaeA*, *fimC*, *fyuA*, *irp2*, *sitA*, and *stx2*, and those of B1 were higher in genes *astA*, *colV*, *hlyA*, *hlyF*, *iss*, *ompT*, and *tsh*. For *ler* and *LT*, the predominant groups were located in D and Clade I, respectively.

**Figure 8:**
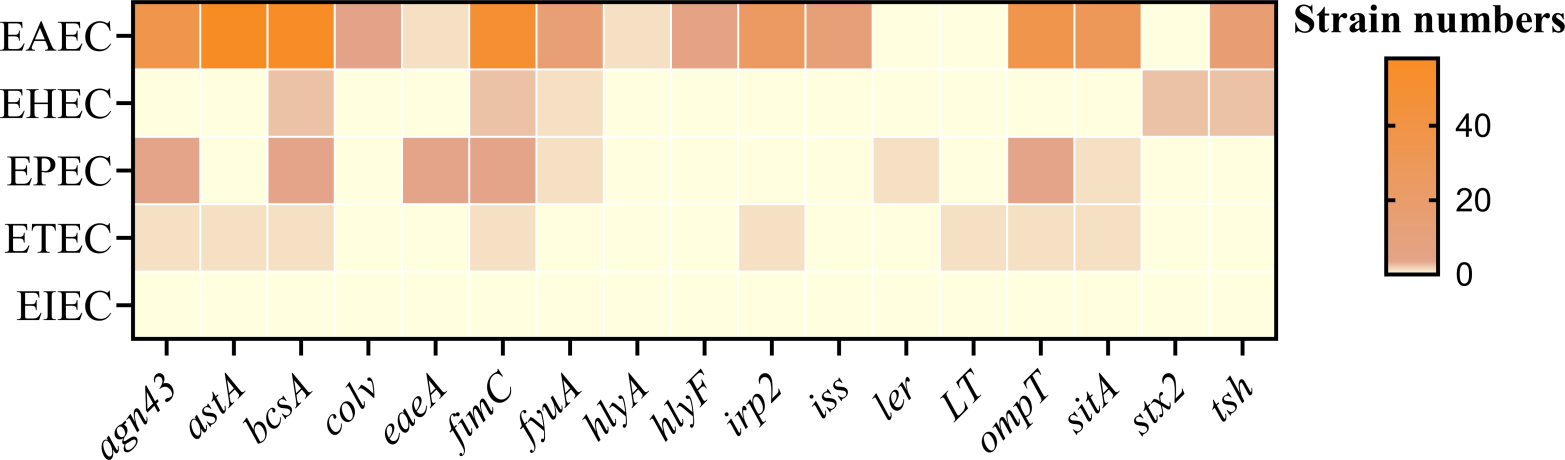
Distribution of virulence genes in DEC.

A total of 64 strains were screened and identified as DEC, the most predominant pathotype belonged to enteroaggregative *E. coli* (EAEC), accounting for 90.63% (58/64). Followed by enteropathogenic *E. coli* (EPEC), enterohemorrhagic *E. coli* (EHEC), and enterotoxigenic *E. coli* (ETEC), accounting for 4.69% (3/64), 3.12% (2/64), 1.56% (1/64), respectively. No enteroinvasive *E. coli* (EIEC) were identified in this study. Our results also revealed that EAEC strains (EAEC5-12) harbored various virulence genes relating to biofilm formation with a higher detection rate of virulence genes (**Figure 8**).

Among the 64 DEC isolates, most isolates exhibited moderate or weak biofilm-forming ability. Namely, 4.69% (3/64), 21.88% (14/64), 59.38% (38/64), and 14.06% (9/64) were classed as strong, moderate, weak, and absent producers of biofilm.

ERIC patterns based on molecular weight and markers were observed. Isolates with one to three similar or different band patterns were grouped, while those with differences in more than three bands were classified into separate types. Based on 61.2% similarity among strains, dendrograms grouped the 64 DEC isolates into five clusters (I, II, III, IV, and V). Of these, 84.38% were in cluster II, where all strong biofilm-forming strains were located (**Table 2**, **Supplementary Figure 2**).

**Table 2:** Biofilm-forming ability and hierarchical clustering 64 DEC.

Clusters	Biofilm-forming ability			
	Strong	Moderate	Weak	Absent
I	0	0	3	1
II	3	14	28	8
III	0	0	2	0
IV	0	0	4	0
V	0	0	1	0
	3 (4.69%)	14 (21.88%)	38 (59.38%)	9 (14.06%)

### 3.5 Whole-genome sequencing

**Figure 9:**
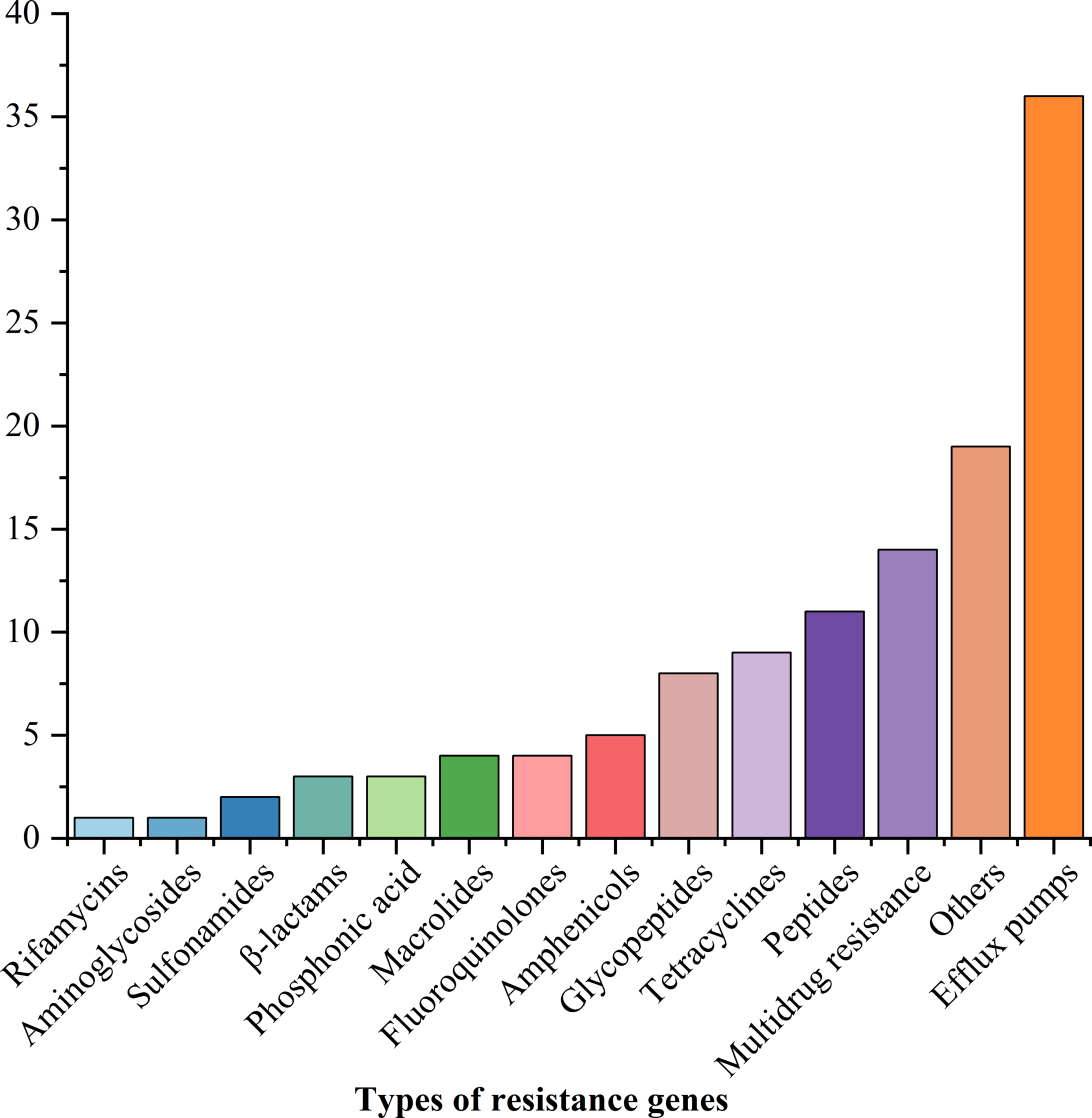
Predicted mechanisms of action for drug resistance genes.

The detected antibiotic resistance profiles for EAEC5–12 largely agreed with the MIC results. Based on the verification against the NCBI AMRFinderPlus database, a total of 120 ARGs were ultimately selected, including those for tetracyclines, aminoglycosides, fluoroquinolones, glycopeptides, and macrolides, 71.67% were involved in antibiotic efflux, 14.17% were involved in antibiotic target alteration, and 15.16% were involved in multiple resistance mechanisms simultaneously (**Figure 9**).

**Figure 10:**
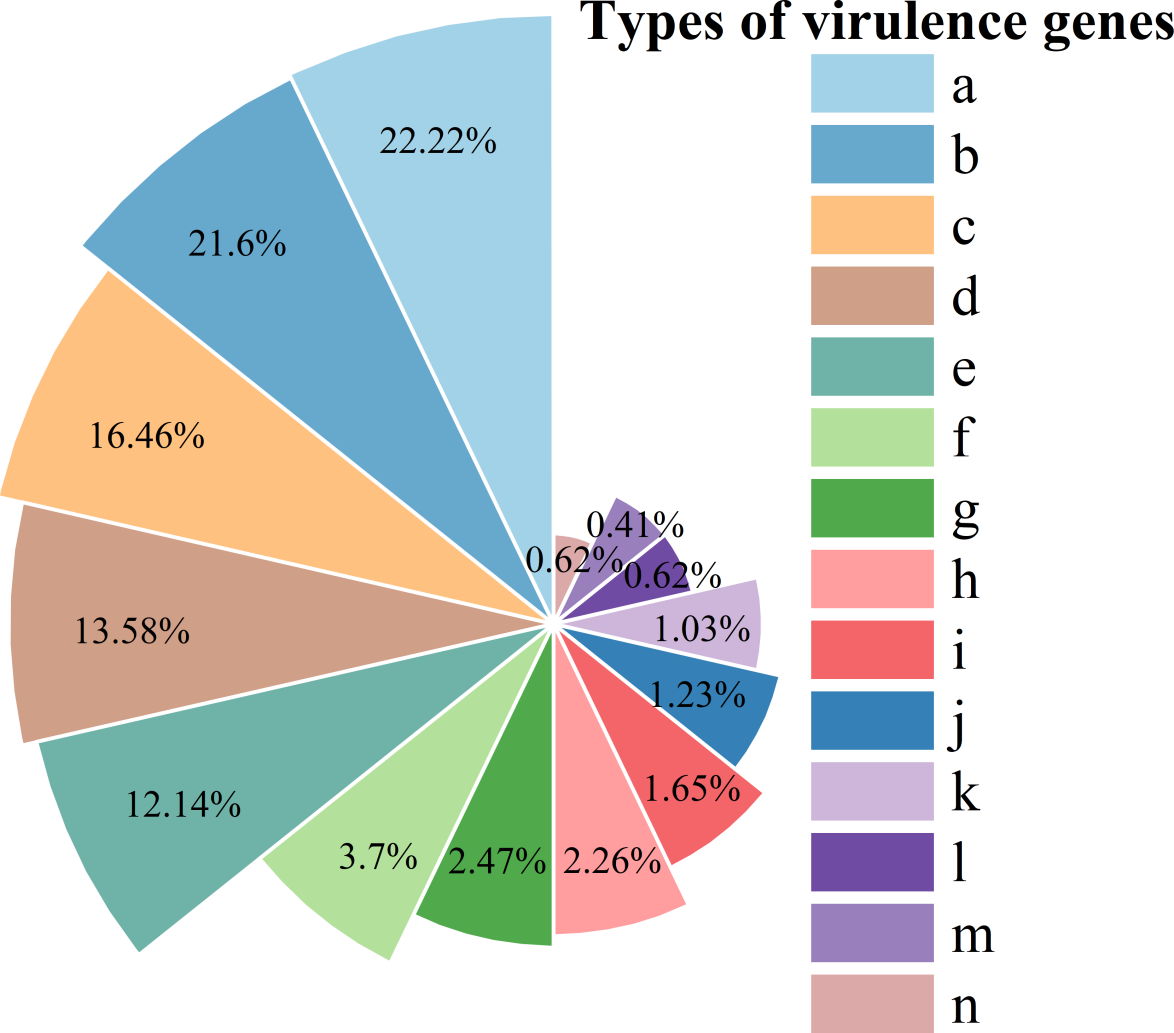
Classification of virulence genes. **(a)** adherence; **(b)** nutritional/metabolic factor; **(c)** immune modulation; **(d)** effector delivery system; **(e)** motility; **(f)** regulation; **(g)** antimicrobial activity/competitive advantage; **(h)** biofilm; **(i)** stress survival; **(j)** exotoxin; **(k)** others; **(l)** exoenzyme; **(m)** invasion; **(n)** post-translational modification.

Gene function annotations for EAEC 5–12 virulence genes showed 486 annotated virulence genes, including functions in adherence, antimicrobial activity/competitive advantage, biofilm formation, effector delivery systems, and others (**Figure 10**).

## 4 Discussion

Bacterial resistance is a growing global issue, with MDR problems becoming increasingly severe. Economic and regulatory challenges contribute to the high prevalence of MDR bacteria in some developing countries. Given the limited research on antimicrobial resistance in Tibetan pigs, in this study, we observed a higher prevalence of multidrug-resistant *E. coli* with virulence factors in Tibetan pigs and the environmental samples (soil and drain water), providing a basis for comparison with reports from other regions and animal populations.

In this study, 84.43% of the 244 *E. coli* isolates from Tibetan were MDR and were resistant to 23 antibiotics with high resistance to sulfonamides, amphenicols, tetracyclines, and *β*-lactams, emphasizing the urgent need to improve drug management and avoid unnecessary antibiotic use in Tibetan pig farms. The result is aligned with a study in Bangladesh, where 98% of the tested strains were MDR (Jain et al., 2021) and another report indicates that resistance rates vary significantly by region and sample types (An, 2024).

Tibetan pig farming is a key economic sector in Garze Tibetan Autonomous Prefecture, Sichuan Province. According to the drug use information provided by the farmers, Ampicillin, Florfenicol, and Doxycycline are the most commonly used antibiotics in Tibetan pig farming due to their low cost and effectiveness. Data indicates that over 75% of tetracycline antibiotics or their metabolites enter the environment through waste, increasing selective pressure and promoting antibacterial resistance (Xu et al., 2021; Larsson and Flach, 2022). In early livestock practices, sulfonamides were often combined with *β*-lactams and tetracyclines (Sánchez-Osuna et al., 2018). Compared with other antibiotics, tetracyclines and sulfonamides are more readily adsorbed by soil and other natural organic matter (Kumar et al., 2005). Thus, surveillance is urgent to avoid the dissemination of antibiotic resistance in Garze Tibetan Autonomous Prefecture, Sichuan Province, China.

Bacterial resistance arises from multiple factors, necessitating a comprehensive, multifaceted approach to understanding its spread. The mobility of resistance genes enables their transfer between bacterial species via mobile genetic elements, facilitating the spread of pathogenic resistance genes (Mmh et al., 2021). Integrases are one of the major causes of multidrug resistance in gram-negative bacteria. Strains with class I integrase exhibit distinct resistance profiles compared to those lacking class I integrase (Sunde and Norström, 2006). We detected 41 ARGs associated with tetracyclines, *β*-lactams, aminoglycosides, fluoroquinolones, amphenicols, and sulfonamides, and 25 were positively detected in this study. Detection rates of ARGs were similar to a previous study, where resistance gene detection rates for *β*-lactams, aminoglycosides, tetracyclines, amphenicols, macrolides, sulfonamides, and polymyxins ranged from 50% to 96% (Peng et al., 2021). A study in India also found that isolates from various animals and their handlers carry multiple resistance genes (Mitra et al., 2024). Integrase genes*, intl1* and *intl2*, have also been detected in pigs, chickens, and cattle (Wang et al., 2019; Yang et al., 2020). We also detected integrase genes, *intl1* and *intl2*, with detection rates of 90.16% and 15.16%, respectively. Our results revealed a strong correlation between resistance genes and resistance phenotypes, with a significant difference between integrase gene-positive and gene-negative strains, highlighting the necessity to monitor integrase genes to prevent worsening antimicrobial resistance in Tibetan pigs.

In this experimental design, we detected 23 virulence genes, the results showed that 17 virulence genes (*agn43*, *astA*, *bcsA*、*colV*, *eaeA*, *fimC*, *fyuA*, *hlyA*, *hlyF*, *irp2*, *iss*, *ler*, *LT*, *ompT*, *sitA*, *stx2*, *tsh*) were positively detected, while 6 genes (*afa*, *bcsB*, *ehxA*, *papC*, *stx1*, and *Vat*) were not detected. Overall, our results indicate that most *E. coli* isolates are diarrheagenic pathotypes. Nevertheless, the occurrence of single or multiple virulence factors does not essentially signify its pathogenicity. Further studies via animal models or tissue cultures are needed to confirm the pathogenicity based on the observed virulence genotypes. Among the detected virulence genes, *bcsA*, *fimC*, and *agn43* had the highest detection rates, with the *agn43*/*bcsA*/*fimC* combination being the most prevalent genotype, accounting for 17.92% (19/106). *Agn43* encodes an adhesin that bacterial attachment to host cells (Sora et al., 2021). *BcsA* encodes bacterial cellulose synthase, which aids in biofilm formation and promotes adherence to host cells (Castiblanco and Sundin, 2018). *FimC* encodes the Type I flagellum adhesin, an adhesion factor critical for fimbriae synthesis; mutations in *fimC* can impair fimbriae formation (Gahlot et al., 2022). The above three virulence genes are all associated with biofilm formation, suggesting that *E. coli* isolated from Tibetan pigs may exert pathogenic effects through biofilm formation. The relationship between biofilm formation and virulence gene expression is needed in our future work.

Sixty-four strains were identified as DEC, with EAEC showing the highest isolation rate. Most of the 17 virulence genes were found in EAEC, likely due to its high prevalence. Phylogenetic analysis showed that the 17 detected virulence genes are mainly distributed in groups A and B1, aligning with Rehman et al.’s finding that virulence genes are predominantly found in group A (Rehman et al., 2017). Notably, studies have found that human-derived *E. coli* in group B2 is associated with the virulence genes *fimH*, *irp2*, *kpsMTII* (Monroy-Pérez et al., 2020). The discrepancies in these findings may be attributed to animal groups or geographical origin.

In this study, we developed a rapid and simple method to investigate the relationship between ERIC-PCR typing and biofilm formation. ERIC-PCR typing identified 11 separating DNA fragments of varying sizes ranging from 300 bp to 2000 bp. Based on a genetic similarity of 61.2%, 84.38% of the DEC were classified as cluster II, with all five clusters showing weak biofilm-forming ability. A study in Iran also found that DNA fragments of varying sizes of 115 tested strains ranged from 380 bp to 3280 bp, with different band distributions depending on the animal source: chicken samples primarily concentrated around 2800 bp, while sheep and cattle samples focused around 1200 bp (Ranjbar et al., 2017).

A total of 120 ARGs were predicted from CARD in EAEC 5-12, including genes for tetracyclines (*tetA*, *tetB*, *tetD*, and *tetT*), aminoglycosides (*aadA5*, aph *(3’’)-Ib*, and *aph(6)-Id*), fluoroquinolones (*mfd*, *gyrA*, and *gyrB*), amphenicols (*cat1*, *cmlV*, and *mexN*), *β*-lactams (*CMY-63*, *mecC*, and *ompK37*), and sulfonamides (*sul2* and *sul3*). Additionally, resistance genes related to efflux pumps, macrolides, glycopeptides, and lacosamide were also predicted, and most were associated with efflux pumps. Although EAEC 5–12 is sensitive to PLB, it is predicted to carry the *MCR-3* gene.

Adhesins are key proteins that facilitate pathogen-host binding, and the genes (*fimA*, *fimB*, *fimC*, etc.) carried by EAEC 5–12 play a role in the pathogen’s colonization process. The *entA*, *entB*, and *fepA* genes in EAEC 5–12 are nutritional/metabolic factors that supply essential nutrients to the pathogen. The *ompA*, *LPS*, and *pbpG* genes are involved in immune regulation, modulating the immune response to the pathogen. Through WGS and VFDB alignment analysis, fourteen virulence genes were predicted in EAEC 5-12, primarily including adhesins, nutritional/metabolic factors, and immune regulators. Most of the virulence genes mentioned above are linked to biofilm formation and pathogenicity, suggesting that effectively inhibiting biofilm formation could be a strategy to reduce pathogenicity in bacterial infections.

A limitation of this study is the small sample size, and our future work should increase sample sizes for WGS. Another important issue to address is that a systematic and comprehensive reference for the research on antimicrobial resistance based on machine learning methods and data mining techniques is urgent in our future work.

## 5 Conclusion

The present study highlights the important role of the Tibetan pigs as a potential reservoir of multidrug-resistant *E. coli* carrying a variety of virulence genes that evoke public health problems by spreading into the environment. The association between antibiotic resistance and virulence genes reveals that virulence characteristics in Tibetan pigs might be selected by antibiotic usage in Tibetan pig farms. In summary, it is urgent to enhance surveillance response systems for monitoring the rational use of antimicrobial agents in Tibetan pigs in Garze Tibetan Autonomous Prefecture, Sichuan province, China.

## Data Availability

The raw data supporting the conclusions of this article will be made available by the authors, without undue reservation.
