# Supplementary material for: Antibiotic resistance, virulence gene, phylogenetic group and genetic diversity of Escherichia coli isolated from Tibetan pig farms in Garze Tibetan Autonomous Prefecture, Sichuan, China
Source: Front Cell Infect Microbiol. 2025 May 12;15:1526028. doi: 10.3389/fcimb.2025.1526028 (PMC12123207; doi:10.3389/fcimb.2025.1526028)
Supplement: Supplementary file 1 [file DataSheet1.docx]

Supplementary Material

**Antibiotic resistance, virulence gene, phylogenetic group and genetic diversity of *Escherichia coli* isolated from Tibetan pigs in Garze Tibetan Autonomous Prefecture, Sichuan, China**

**Xing Zhao ^1^†, Jun Liang ^2^†, Zhaobin Xia ^1^and Chaoxi Chen ^1^***

^1^ College of Animal and Veterinary Sciences, Southwest Minzu University, Chengdu, Sichuan, China

^2^ Zhengzhou Inspection and Testing Center of Products Quality, Zhengzhou 450001, China

*** Correspondence:** chaoxi8832@163.com

**†These authors have contributed equally to this work**

**Supplementary Table 1** Sampling information

| **Sampling sites** | **Sample type** | **Sampling time** |
| --- | --- | --- |
| Shangkuiwu Village, Dewei Town, Luding County | Feces (37), Water (5), Soil (10) | June 19, 2022 |
| Mohegou Village, Dewei Town, Luding County | Feces (14), Water (0), Soil (0) | June 19, 2022 |
| Zhengdou Village, Shagong Town, Xiangcheng County | Feces (27), Water (0), Soil (3) | June 29, 2022 |
| Lamu Village, Mula Town, Daocheng County | Feces (95), Water (2), Soil (7) | June 30, 2022 |
| Zhongdui Village, Chitu Town, Daocheng County | Feces (22), Water (0), Soil (0) | July 1, 2022 |
| Mushan Village, Luqiao Town, Luding County | Feces (11), Water (0), Soil (0) | October 5, 2022 |
| Jianchaping Village, Lengmoi Town, Luding County | Feces (11), Water (0), Soil (0) | October 6, 2022 |

**Supplementary Table 2** Primer sequences of antibacterial resistance genes and integrase genes

| **Types of antibiotics** | **Gene names** | **Primer sequence (5’→3’)** | **Fragment length (bp)** | **Annealing temperature (°C)** | **References/Accession No.** |
| --- | --- | --- | --- | --- | --- |
| Tetracyclines | *tetA* | F: CACTATGGCATTCTGCTGGC | 948 | 60 | X00006 |
|  |  | R: CATAGATCGCCGTGAAGAGG |  |  |  |
|  | *tetB* | F: GCCCAGTGCTGTTGTTGTC | 553 | 60 | J01830 |
|  |  | R: AAGACCAAGACCCGCTAATG |  |  |  |
|  | *tetC* | F: TCCTGCTCGCTTCGCTACT | 730 | 58 | AB089598 |
|  |  | R: TGGTCGTCATCTACCTGC |  |  |  |
|  | *tetD* | F: AAACCATTACGGCATTCTGC | 787 | 56 | L06798 |
|  |  | R: GACCGGATACACCATCCATC |  |  |  |
|  | *tetE* | F: AAACCACATCCTCCATACGC | 278 | 55 | L06940 |
|  |  | R: AAATAGGCCACAACCGTCAG |  |  |  |
|  | *tetG* | F: CGGTCTTATGGGTGCTCTA | 721 | 58 | AF071555 |
|  |  | R: CCTTGCTTGTTACTGAC |  |  |  |
|  | *tetK* | F: TCGATAGGAACAGCAGTA | 169 | 53 | S67449 |
|  |  | R: CAGCAGATCCTACTCCTT |  |  |  |
|  | *tetL* | F: TCGTTAGCGTGCTGTCATTC | 267 | 55 | U17153 |
|  |  | R: GTATCCCACCAATGTAGCCG |  |  |  |
|  | *tetM* | F: TTATCAACGGTTTATCAGG | 397 | 55 | (Beeton et al., 2009) |
|  |  | R: CGTATATATGCAAGACG |  |  |  |
|  | *tetO* | F: AACTTAGGCATTCTGGCTCAC | 515 | 55 | Y07780 |
|  |  | R: TCCCACTGTTCCATATCGTCA |  |  |  |
|  | *tetX* | F: CAATAATTGGTGGTGGACCC | 468 | 56 | M37699 |
|  |  | R: TTCTTACCTTGGACATCCCG |  |  |  |
| *β-*lactams | *bla*_CMY-2_ | F: ATGATGAAAAAATCGTTATGC | 1200 | 57 | (Akunda et al., 2023) |
|  |  | R: TTGCAGCTTTTCAAGAATGCGC |  |  |  |
|  | *bla*_CTX-M-U_ | F: ATGTGCAGTACCAGTAAAGT | 593 | 52 | (Li, 2019) |
|  |  | R: TGGGTRAAGTARGTCACCAGAA |  |  |  |
|  | *bla*_DHA_ | F: AACTTTCACAGGTGTGCTGGGT | 405 | 59 | (Singh et al., 2019) |
|  |  | R: CCGTACGCATACTGGCTTTGC |  |  |  |
|  | *bla*_MOX_ | F: GCTGCTCAAGGAGCACAGGAT | 520 | 58 | (Zhong, 2013) |
|  |  | R: CACATTGACATAGGTGTGGTGC |  |  |  |
|  | *bla*_NDM_ | F: GGTTTGGCGATCTGGTTTTC | 621 | 55 | (Li, 2019) |
|  |  | R: CGGAATGGCTCATCACGATC |  |  |  |
|  | *bla*_OXA_ | F: GGCACCAGATTCAACTTTCAAG | 564 | 55 | GQ896556 |
|  |  | R: GACCCCAAGTTTCCTGTAAGTG |  |  |  |
|  | *bla*_SHV_ | F: CACTCAAGGATGTATTGTG | 885 | 53 | (Liu et al., 2018) |
|  |  | R: TTAGCGTTGCCAGTGCTCG |  |  |  |
|  | *bla*_TEM_ | F: ATAAAATTCTTGAAGACGAAA | 1150 | 53 | (Afsharnia et al., 2018) |
|  |  | R: GACAGTTACCAATGCTTAATCA |  |  |  |
| Aminoglycosides | *aac(3’)-Ⅱ* | F: ACTGTGATGGGATACGCGTC | 237 | 58 | (Sáenz et al., 2004) |
|  |  | R: CTCCGTCAGCGTTTCAGCTA |  |  |  |
|  | *aac(3’)-Ⅳ* | F: GGCCACTTGGACTGATCGAG | 409 | 58 | X01385 |
|  |  | R: GCGGATGCAGGAAGATCAAC |  |  |  |
|  | *aadA2* | F: GGTGCTAAGCGTCATTGAGC | 470 | 57 | AB154408 |
|  |  | R: GCTTCAAGGTTTCCCTCAGC |  |  |  |
|  | *ant(3’)-Ia* | F: ATCTGGCTATCTTGCTGACA | 284 | 53 | (Zhang et al., 2018) |
|  |  | R: TATGACGGGCTGATACTGG |  |  |  |
|  | *aph(3’)-Ⅶ* | F: TCCACAGGATGGCAAGATCC | 690 | 55 | AY260546 |
|  |  | R: TTCAACGGGAAACGTCTTGC |  |  |  |
|  | *rmtB* | F: ACATCAACGATGCCCTCAC | 472 | 53 | AB103506 |
|  |  | R: AAGTTCTGTTCCGATGGTC |  |  |  |
| Quinolones | *aac(6’)-Ib-cr* | F: TTGCGATGCTCTATGAGTGGCTA | 482 | 56 | (El-Badawy et al., 2017) |
|  |  | R: CTCGAATGCCTGGCGTGTTT |  |  |  |
|  | *qepA* | F: GCAGGTCCAGCAGCGGGTAG | 199 | 56 | (Park et al., 2022) |
|  |  | R: CTTCCTGCC CGAGTATCGTG |  |  |  |
|  | *qnrA* | F: TCAGCAAGAGGATTTCTCA | 627 | 55 |  |
|  |  | R: GGCAGCACTATTACTCCCA |  |  |  |
|  | *qnrB* | F: ACGATGCCTGGTAGTTGTCC | 469 | 55 |  |
|  |  | R: ACGACATTCGTCAACTGCAA |  |  |  |
|  | *qnrC* | F: GGGTTGTACATTTATTGAATC | 447 | 48 | (Wu, 2018) |
|  |  | R: TCCACTTTACGAGGTTCT |  |  |  |
|  | *qnrD* | F: CGAGATCAATTTACGGGGAATA | 582 | 53 |  |
|  |  | R: AACAAGCTGAAGCGCCTG |  |  |  |
|  | *qnrS* | F: ACGACATTCGTCAACTGCAA | 417 | 55 | (Mabika Mabika et al., 2021) |
|  |  | R: TAAATTGGCACCCTGTAGGC |  |  |  |
| Amphenicols | *cat1* | F: CTTGTCGCCTTGCGTATAAT | 508 | 53 | (Lin et al., 2023) |
|  |  | R: ATCCCAATGGCATCGTAAAG |  |  |  |
|  | *cat2* | F: AACGGCATGATGAACCTGAA | 547 | 53 | (Shivakumaraswamy et al., 2019) |
|  |  | R: ATCCCAATGGCATCGTAAAG |  |  |  |
|  | *cfr* | F: TAAGAATAATAATGAGC | 518 | 43 | (Kun, 2019) |
|  |  | R: TATAGAAAGTCTACGAGG |  |  |  |
|  | *cmlA* | F: CGCCACGGTGTTGTTGTTAT | 394 | 59 | (Chen et al., 2004) |
|  |  | R: GCGACCTGCGTAAATGTCAC |  |  |  |
|  | *cmlB* | F: ACTCGGCATGGACATGTACT | 840 | 57 |  |
|  |  | R: ACGGACTGCGGAATCCATAG |  |  |  |
|  | *floR* | F: CTGAGGGTGTCGTCATCTAC | 673 | 54 |  |
|  |  | R: GCTCCGACAATGCTGACTAT |  |  |  |
| Sulfonamides | *sul1* | F: TCAGACGTCGTGGATGTCG | 346 | 57 | (Khorsi et al., 2015) |
|  |  | R: CGAAGAACCGCACAATCTCG |  |  |  |
|  | *sul2* | F: CCTGTTTCGTCCGACACAGA | 435 | 59 | (Shivakumaraswamy et al., 2019) |
|  |  | R: GAAGCGCAGCCGCAATTCAT |  |  |  |
|  | *sul3* | F: AGATGTGATTGATTTGGGAGC | 443 | 53 | (Manzoor et al., 2023) |
|  |  | R: TAGTTGTTTCTGGATTAGAGCCT |  |  |  |
| Integrase | *intl1* | F: CCTCCCGCACGATGATC | 280 | 57 | (Ma et al., 2019) |
|  |  | R: TCCACGCATCGTCAGGC |  |  |  |
|  | *intl2* | F: TTATTGCTGGGATTAGGC | 233 | 52 | (Rezanejad et al., 2019) |
|  |  | R: ACGGCTACCCTCTGTTATC |  |  |  |

**Supplementary Table 3** Primer sequences of virulence genes

| **Gene categories** | **Gene names** | **Primer sequence (5’→3’)** | **Fragment length (bp)** | **Annealing temperature (°C)** | **References/Accession No.** |
| --- | --- | --- | --- | --- | --- |
| Adherence | *afa* | F: GGCAGAGGGCCGGCAACAGGC | 559 | 65 | (Johnson and Stell, 2000) |
|  |  | R: CCCGTAACGCGCCAGCATCTC |  |  |  |
|  | *fimC* | F: GGAAATAACATTCTGCTTGC | 288 | 48 | (Jeong et al., 2012) |
|  |  | R: TTTGTTGCATCAAGAATACG |  |  |  |
|  | *papC* | F: GACGGCTGTACTGCAGGGTGTGGCG | 328 | 55 | (Zhang et al., 2018) |
|  |  | R: ATATCCTTTCTGCAGGGATGCAATA |  |  |  |
| Biofilm | *agn43* | F: GACTATGACCGGATTSTGGCAGGCT | 499 | 61 | U24429 |
|  |  | R: GTGGCTCCAGCATCARTTGTCAG |  |  |  |
| Cellulose structure | *bcsA* | F: GATTTTCGACTGCGACCACGTS | 185 | 54 | (Hu et al., 2015) |
|  |  | R: ACATGTCGTTRCCRTCCTGCAC |  |  |  |
|  | *bcsB* | F: GCAGATTTTCCGYCTCGAYAAC | 388 | 55 |  |
|  |  | R: CSACRTACTCTTCTTCCACCAG |  |  |  |
| Colicin V | *colv* | F: TGGTAGAATTGTGCCAGAGCAAG | 1180 | 57 | (Mohamed et al., 2014) |
|  |  | R: GAGCTGTTTGTAGCGAAGCC |  |  |  |
| Effector delivery system | *tsh* | F: ACTATTCTCTGCAGGAAGTC | 824 | 52 | AF218073 |
|  |  | R: CTTCCGATGTTCTGAACGT |  |  |  |
|  | *vat* | F: TCCTGGGACATAATGGCTAG | 981 | 51 | (Jeong et al., 2012) |
|  |  | R: GTGTCAGAACGGAATTGT |  |  |  |
| LEE virulence island | *eaeA* | F: ATGCTTAGTGCTGGTTTAGG | 248 | 52 | EF079676 |
|  |  | R: GCCTTCATCATTTCGCTTTC |  |  |  |
|  | *ler* | F: CGCACACAACAAGCCCATAC | 196 | 57 | AF200363 |
|  |  | R: GATGAGTTCCGGCGAGCAA |  |  |  |
| Nutritional/metabolic factor | *fyuA* | F: TGATTAACCCCGCGACGGGAA | 880 | 59 | Z38064 |
|  |  | R: CGCAGTAGGCACGATGTTGTA |  |  |  |
|  | *irp2* | F: AAGGATTCGCTGTTACCGGAC | 280 | 59 | (Jeong et al., 2012) |
|  |  | R: TCGTCGGGCAGCGTTTCTTCT |  |  |  |
|  | *sitA* | F: AGGGGGCACAACTGATTCTCG | 608 | 60 | (Gazal et al., 2015) |
|  |  | R: TACCGGGCCGTTTTCTGTGC |  |  |  |
| Outer membrane protein | *ompT* | F: ATCTAGCCGAAGAAGGAGGC | 559 | 55 | (Jeong et al., 2012) |
|  |  | R: CCCGGGTCATAGTGTTCATC |  |  |  |
| Serum resistance protein | *iss* | F: CAGCAACCCGAACCACTTGATG | 323 | 60 | (Johnson et al., 2008) |
|  |  | R: AGCATTGCCAGAGCGGCAGAA |  |  |  |
| Toxins | *astA* | F: TGCCATCAACACAGTATATCC | 111 | 59 | (Wilczyński et al., 2022) |
|  |  | R: TAGGATCCTCAGGTCGCGAGTGACGGC |  |  |  |
|  | *ehxA* | F: CAATAATTGGTGGTGGACCC | 583 | 53 | EF088504 |
|  |  | R: TTCTTACCTTGGACATCCCG |  |  |  |
|  | *hlyA* | F: AACAAGGATAAGCACTGTTCTGGCT | 1177 | 59 | (Ruiz et al., 2002) |
|  |  | R: ACCATATAAGCGGTCATTCCCGTCA |  |  |  |
|  | *hlyF* | F: GGCGATTTAGGCATTCCGATACTC | 599 | 58 | (Jeong et al., 2012) |
|  |  | R: ACGGGGTCGCTAGTTAAGGAG |  |  |  |
|  | *LT* | F: ATGAGTACTTCGATAGAGG | 279 | 50 | (Jeong et al., 2012) |
|  |  | R: ATGGTATTCCACCTAACGC |  |  |  |
|  | *stx1* | F: ACACTGGATGATCTCAGTGG | 614 | 52 | (Wang et al., 2009) |
|  |  | R: CTGAATCCCCCTCCATTATG |  |  |  |
|  | *stx2* | F: CCTGTCAACTGAGCACTTTG | 779 | 55 |  |
|  |  | R: CCATGACAACGGACAGCAGTT |  |  |  |

**Supplementary Table 4** **Primer sequences for diarrheagenic *E. coli* typing, phylogenetic typing, and ERIC-PCR typing**

| **Types of genes** | **Gene names** | **Primer sequence (5’→3’)** | **Fragment length (bp)** | **Annealing temperature (°C)** | **References/ Accession No.** |
| --- | --- | --- | --- | --- | --- |
| Diarrheagenic *E. coli* | *invE* | F: CGATAGATGGCGAGAAATTATATCCCG | 63 | 766 | AF283289.1 |
|  |  | R: CGATCAAGAATCCCTAACAGAAGAATCAC |  |  |  |
|  | *escV* | F: ATTCTGGCTCTCTTCTTCTTTATGGCTG | 63 | 544 | FM180568.1 |
|  |  | R: CGTCCCCTTTTACAA ACTTCATCGC |  |  |  |
|  | *bfpB* | F: GACACCTCATTGCTGAAGTCG | 63 | 910 | FM180569.1 |
|  |  | R: CCAGAACACCTCCGTTATGC |  |  |  |
|  | *stx1* | F: CGATGTTACGGTTTGTTACTGTGACAGC | 63 | 244 | AE005174.2 |
|  |  | R: AATGCCACGCTTCCCAGAATTG |  |  |  |
|  | *stx2* | F: GTTTTGACCATCTTCGTCTGATTATTGAG | 63 | 324 | AE005174.2 |
|  |  | R: AGCGTAAGGCTTCTGCTGTGAC |  |  |  |
|  | *LT* | F: GAACAGGAGGTTTCTGCGTTAGGTG | 63 | 655 | CP000795.1 |
|  |  | R: CTTTCAATGGCTTTTTTTTGGGAGTC |  |  |  |
|  | *stp* | F: CCTCTTTTAGYCAGACARCTGAATCASTTG | 63 | 157 | AJ555214.1/  AY342057.1 |
|  |  | R: CAGGCAGGATTACAACAAAGTTCACAG |  |  |  |
|  | *sth* | F: TGTCTTTTTCACCTTTCGCTC | 63 | 171 | CP000795.1 |
|  |  | R: CGGTACAAGCAGGATTACAACAC |  |  |  |
|  | *aggR* | F: ACGCAGAGTTGCCTGATAAAG | 63 | 400 | Z18751.1 |
|  |  | R: AATACAGAATCGTCAGCATCAGC |  |  |  |
|  | *pic* | F: AGCCGTTTCCGCAGAAGCC | 63 | 1111 | AF097644.1 |
|  |  | R: AAATGTCAGTGAACCGACGATTGG |  |  |  |
|  | *astA* | F: TGCCATCAACACAGTATATCCG | 63 | 102 | AF161001.1 |
|  |  | R: ACGGCTTTGTAGTCCTTCCAT |  |  |  |
| Phylogenetic analysis | *chuA* | F: ATGGTACCGGACGAACCAAC | 59 | 288 | (O et al., 2013) |
|  |  | R: TGCCGCCAGTACCAAAGACA |  |  |  |
|  | *yjaA* | F: CAAACGTGAAGTGTCAGGAG | 59 | 211 |  |
|  |  | R: AATGCGTTCCTCAACCTGTG |  |  |  |
|  | *TspE4.C2* | F: CACTATTCGTAAGGTCATCC | 59 | 152 |  |
|  |  | R: AGTTTATCGCTGCGGGTCGC |  |  |  |
|  | *arpA* | F: AACGCTATTCGCCAGCTTGC | 59 | 400 |  |
|  |  | R: TCTCCCCATACCGTACGCTA |  |  |  |
|  | *arpAgpE* | F: GATTCCATCTTGTCAAAATATGCC | 57 | 301 | (Lescat et al., 2013) |
|  |  | R: GAAAAGAAAAAGAATTCCCAAGAG |  |  |  |
|  | *trpAgpC* | F: AGTTTTATGCCCAGTGCGAG | 59 | 219 |  |
|  |  | R: TCTGCGCCGGTCACGCCC |  |  |  |
|  | *trpBA* | F: CGGCGATAAAGACATCTTCAC | 59 | 489 | (Clermont et al., 2008) |
|  |  | R: GCAACGCGGCCTGGCGGAAG |  |  |  |
| ERIC-PCR typing | - | F: ATGTAAGCTCCTGGGGATTCAC | 53 | / | (Ranjbar et al., 2017) |
|  |  | R: AAGTAAGTGACTGGGGTGAGCG |  |  |  |

**Supplementary Table 5** Criteria for *E. coli* phylogenetic typing

| ***arpA*** | ***chuA*** | ***yjaA*** | ***TspE4.C2*** | **Phylo-Group** | **Next step** |
| --- | --- | --- | --- | --- | --- |
| + | - | - | - | A |  |
| + | - | - | + | B1 |  |
| - | + | - | - | F |  |
| - | + | + | - | B2 |  |
| - | + | + | + | B2 |  |
| - | + | - | + | B2 |  |
| - | - | + | - | Clade Ⅰ |  |
| + | - | + | - | A or C | Screen using C-specific primers (*trpAgpC*). If C+ then C, else A |
| + | + | - | - | D or E | Screen using E-specific primers (*arpAgpE*). If E+ then E, else D  Screen using E-specific primers (*arpAgpE*). If E+ then E, else D |
| + | + | - | + | D or E |  |
| + | + | + | - | E or Clade Ⅰ | Screen using E-specific primers (*arpAgpE*). If E+ then E, else Clade Ⅰ |

**Supplementary Table 6** Reference table for the five target bands and their corresponding types of DEC

| **Categories of DEC** | **Combination of target band types** |  |
| --- | --- | --- |
| EAEC | *aggR*, *astA*, *pic,* one or more of the following genes were positive | *uidA* (+/-) |
| EPEC | *bfpB* (+/-), *escV* (+), *stx1* (-), *stx2* (-) |  |
| STEC/EHEC | *escV* (+/-), *stx1* (+), *stx2* (-), *bfpB* (-) |  |
|  | *escV* (+/-), *stx1* (-), *stx2* (+), *bfpB* (-) |  |
|  | *escV* (+/-), *stx1* (+), *stx2* (+), *bfpB* (-) |  |
| ETEC | *LT*, *stp*, *sth,* one or more of the following genes were positive |  |
| EIEC | *invE* (+) |  |

**Supplementary Table 7** Differences in drug resistance of *E. coli* in different sampling sites

| **Types of antibiotics** | **Names of antibiotics** | ***P* value** |
| --- | --- | --- |
| Tetracyclines | OTC | 0.158 |
|  | DOX | 0.012 |
|  | TCY | 0.001 |
| Sulfonamides | SMZ | 0.789 |
|  | SMX | 0.274 |
| *β* -lactams | AMP | 0.001 |
|  | OXA | 0.351 |
|  | AMC | 0.045 |
|  | TIO | 0.001 |
|  | CEF | 0.001^a^ |
| Amphenicols | FLR | 0.405 |
|  | CHL | ——^b^ |
| Rifamycins | RIF | 0.023 |
| Aminoglycosides | STR | 0.325 |
|  | GEN | 0.001 |
|  | SPT | 0.001 |
|  | KAN | 0.001 |
| Quinolones | NAC | 0.001 |
|  | CIP | 0.033 |
|  | ENR | 0.101 |
|  | SAR | 0.001 |
| Polypeptides | PLB | 0.278 |
| Polyphosphates | FOS | 0.381 |

**Notes:** a: *Fisher*'s exact test; b: Not conform to the algorithm

**Supplementary Table 8** Correlation analysis between antimicrobial resistance genes and phenotypes

| **Types of antibiotics** | **Names of antibiotics** | **Genes** | **Concordance rate (%)** | ***P* value** |
| --- | --- | --- | --- | --- |
| Tetracyclines | OTC | *tetA* | 66.15 | 0.001 |
|  |  | *tetB* | 37.44 | 0.247 |
|  |  | *tetD* | 22.56 | 0.341 |
|  |  | *tetM* | 8.72 | 0.028 ^a^ |
|  | DOX | *tetA* | 72.00 | 0.001 |
|  |  | *tetB* | 44.67 | 0.001 |
|  |  | *tetD* | 26.00 | 0.024 |
|  |  | *tetM* | 10.00 | 0.019 |
|  | TCY | *tetA* | 71.60 | 0.001 |
|  |  | *tetB* | 41.36 | 0.009 |
|  |  | *tetD* | 25.93 | 0.013 |
|  |  | *tetM* | 9.26 | 0.048 |
| Sulfonamides | SMZ | *sul1* | 42.55 | 0.311 ^a^ |
|  |  | *sul2* | 73.62 | 0.001 |
|  |  | *sul3* | 82.98 | 0.058 ^a^ |
|  | SMX | *sul1* | 43.23 | 0.077 |
|  |  | *sul2* | 73.80 | 0.002 ^a^ |
|  |  | *sul3* | 82.97 | 0.156 ^a^ |
| *β* -lactams | AMP | *bla*_CMY-2_ | 15.44 | 0.274 |
|  |  | *bla*_CTX-M-U_ | 47.65 | 0.02 |
|  |  | *bla*_DHA_ | 4.70 | 0.842 |
|  |  | *bla*_OXA_ | 10.74 | 0.838 |
|  |  | *bla*_SHV_ | 42.28 | 0.057 |
|  |  | *bla*_TEM_ | 96.64 | 0.006 |
|  | OXA | *bla*_CMY-2_ | 13.84 | 1 ^a^ |
|  |  | *bla*_CTX-M-U_ | 42.86 | 0.264 |
|  |  | *bla*_DHA_ | 4.91 | 1 ^a^ |
|  |  | *bla*_OXA_ | 12.05 | 0.141 ^a^ |
|  |  | *bla*_SHV_ | 46.43 | 0.462 |
|  |  | *bla*_TEM_ | 93.30 | 0.637 ^a^ |
|  | AMC | *bla*_CMY-2_ | 22.54 | 0.008 |
|  |  | *bla*_CTX-M-U_ | 52.11 | 0.036 |
|  |  | *bla*_DHA_ | 7.04 | 0.339 ^a^ |
|  |  | *bla*_OXA_ | 7.04 | 0.199 |
|  |  | *bla*_SHV_ | 42.25 | 0.328 |
|  |  | *bla*_TEM_ | 95.77 | 0.281 |
|  | TIO | *bla*_CMY-2_ | 28.57 | 0.034 ^a^ |
|  |  | *bla*_CTX-M-U_ | 78.57 | 0.001 |
|  |  | *bla*_DHA_ | 7.14 | 0.634 ^a^ |
|  |  | *bla*_OXA_ | 21.43 | 0.1 ^a^ |
|  |  | *bla*_SHV_ | 39.29 | 0.377 |
|  |  | *bla*_TEM_ | 100.00 | 0.232 ^a^ |
|  | CEF | *bla* _CMY-2_ | 10.00 | 0.63 |
|  |  | *bla*_CTX-M-U_ | 90.00 | 0.001 |
|  |  | *bla*_DHA_ | 5.00 | 1 ^a^ |
|  |  | *bla*_OXA_ | 15.00 | 0.472 ^a^ |
|  |  | *bla*_SHV_ | 35.00 | 0.257 |
|  |  | *bla*_TEM_ | 100.00 | 0.374 ^a^ |
| Amphenicols | FLR | *cat1* | 5.33 | 0.006 |
|  |  | *cat2* | 13.33 | 0.314 |
|  |  | *cmlA* | 80.89 | 0.54 |
|  |  | *floR* | 88.00 | 0.706 |
|  | CHL | *cat1* | 7.00 | 1 |
|  |  | *cat2* | 13.99 | 1 |
|  |  | *cmlA* | 81.48 | 1 |
|  |  | *floR* | 88.48 | 1 |
| Aminoglycosides | STR | *aac(3’)-Ⅱ* | 89.23 | 0.377 |
|  |  | *aadA2* | 73.85 | 0.439 |
|  |  | *ant(3’)-Ia* | 90.77 | 0.379 ^a^ |
|  |  | *aph(3’)-Ⅶ* | 70.77 | 0.637 |
|  | GEN | *aac(3’)-Ⅱ* | 100.00 | 0.086 ^a^ |
|  |  | *aadA2* | 83.87 | 0.073 |
|  |  | *ant(3’)-Ia* | 93.55 | 1 ^a^ |
|  |  | *aph(3’)-Ⅶ* | 70.97 | 0.746 |
|  | SPT | *aac(3’)-Ⅱ* | 90.22 | 0.482 |
|  |  | *aadA2* | 89.13 | 0.001 |
|  |  | *ant(3’)-Ia* | 97.83 | 0.031 |
|  |  | *aph(3’)-Ⅶ* | 77.17 | 0.022 |
|  | KAN | *aac(3’)-Ⅱ* | 97.22 | 0.324 ^a^ |
|  |  | *aadA2* | 80.56 | 0.137 |
|  |  | *ant(3’)-Ia* | 97.22 | 0.479 ^a^ |
|  |  | *aph(3’)-Ⅶ* | 94.44 | 0.001 |
| Quinolones | NAL | *aac(6’)-Ib-cr* | 13.46 | 0.322 |
|  |  | *qnrA* | 0.00 | 1 ^a^ |
|  |  | *qnrD* | 25.00 | 0.061 |
|  |  | *qnrS* | 80.77 | 0.214 |
|  | CIP | *aac(6’)-Ib-cr* | 10.34 | 1 ^a^ |
|  |  | *qnrA* | 0.00 | 1 ^a^ |
|  |  | *qnrD* | 41.38 | 0.526 |
|  |  | *qnrS* | 79.31 | 0.259 ^a^ |
|  | ENR | *aac(6’)-Ib-cr* | 8.89 | 1 ^a^ |
|  |  | *qnrA* | 0.00 | 1 ^a^ |
|  |  | *qnrD* | 37.78 | 0.791 |
|  |  | *qnrS* | 82.22 | 0.41 |
|  | SAR | *aac(6’)-Ib-cr* | 10.20 | 1 ^a^ |
|  |  | *qnrA* | 0.00 | 1 ^a^ |
|  |  | *qnrD* | 34.69 | 0.823 |
|  |  | *qnrS* | 83.67 | 0.589 |

**Notes:** a: *Fisher*'s exact test

**Supplementary Table 9** Correlation analysis between antibiotic resistance genes and integrase genes

| **Types of genes** | **Names of genes** | ***intl1*** | | ***intl2*** | |
| --- | --- | --- | --- | --- | --- |
|  |  | **Consistency rate (%)** | ***P* value** | **Consistency rate (%)** | ***P* value** |
| Tetracyclines | *tetA* | 90.97 | 0.611 | 18.75 | 0.061 |
|  | *tetB* | 96.55 | 0.013 | 16.09 | 0.764 |
|  | *tetD* | 88.46 | 0.642 | 23.08 | 0.073 |
|  | *tetM* | 100.00 | 0.388 ^a^ | 17.65 | 0.728 ^a^ |
| *β* -lactams | *bla*_CMY-2_ | 96.97 | 0.216 ^a^ | 33.33 | 0.002 |
|  | *bla*_CTX-M-U_ | 96.08 | 0.216 ^a^ | 21.57 | 0.018 |
|  | *bla*_DHA_ | 91.67 | 1 ^a^ | 50.00 | 0.004 ^a^ |
|  | *bla*_OXA_ | 100.00 | 0.086 ^a^ | 7.41 | 0.391 |
|  | *bla*_SHV_ | 93.04 | 0.154 | 24.35 | 0.001 |
|  | *bla*_TEM_ | 93.39 | 0.001 ^a^ | 15.86 | 0.482 ^a^ |
| Aminoglycosides | *aac(3’)-Ⅱ* | 90.63 | 0.427 ^a^ | 15.18 | 0.001 |
|  | *aadA2* | 91.81 | 0.186 | 16.37 | 0.42 |
|  | *ant(3’)-Ia* | 90.79 | 0.199 ^a^ | 16.23 | 0.141 ^a^ |
|  | *aph(3’)-Ⅶ* | 95.21 | 0.001 | 14.37 | 0.611 |
| Quinolones | *aac(6’)-Ib-cr* | 100.00 | 0.143 ^a^ | 16.67 | 0.768 |
|  | *qnrA* | 0.00 | 0.098 ^a^ | 0.00 | 1 ^a^ |
|  | *qnrD* | 93.18 | 0.234 | 29.55 | 0.001 |
|  | *qnrS* | 91.90 | 0.055 ^a^ | 17.14 | 0.001 |
| Amphenicols | *cat1* | 94.12 | 1 ^a^ | 17.65 | 0.728 ^a^ |
|  | *cat2* | 88.24 | 0.755 ^a^ | 20.59 | 0.342 |
|  | *cmlA* | 92.46 | 0.022 ^a^ | 16.08 | 0.401 |
|  | *floR* | 92.59 | 0.002 ^a^ | 16.20 | 0.271 ^a^ |
| Sulfonamides | *sul1* | 96.08 | 0.009 | 19.61 | 0.101 |
|  | *sul2* | 90.80 | 0.596 | 14.37 | 0.585 |
|  | *sul3* | 92.50 | 0.021 ^a^ | 17.50 | 0.03 |

**Notes:** a: *Fisher*'s exact test

**Supplementary Table 10** Virulence genotypes of 244 *E. coli*

| **Virulence** **genotypes** | **No.** |
| --- | --- |
| *agn43/astA/bcsA/colv/eaeA/fimC/hlyf/iss/ompT/sitA/tsh* | 1 |
| *agn43/astA/bcsA/colv/fimC/fyuA/hlyf/irp2/iss/ompT/sitA* | 2 |
| *agn43/astA/bcsA/colv/fimC/hlyf/irp2/iss/ompT/sitA/tsh* | 1 |
| *agn43/bcsA/colv/fimC/fyuA/hlyf/irp2/iss/ompT/sitA/tsh* | 1 |
| *agn43/astA/bcsA/fimC/hlyf/irp2/iss/ompT/sitA/tsh* | 2 |
| *agn43/bcsA/fimC/fyuA/hlyA/hlyf/iss/ompT/sitA/tsh* | 1 |
| *agn43/astA/bcsA/fimC/fyuA/irp2/iss/ompT/sitA* | 1 |
| *agn43/astA/bcsA/fimC/fyuA/irp2/ompT/sitA/tsh* | 1 |
| *agn43/bcsA/colv/fimC/fyuA/irp2/iss/ompT/sitA* | 1 |
| *agn43/bcsA/colv/fimC/hlyf/iss/ompT/sitA/tsh* | 1 |
| *agn43/bcsA/fimC/hlyA/hlyf/iss/ompT/sitA/tsh* | 1 |
| *agn43/astA/bcsA/fimC/irp2/iss/ompT/sitA* | 1 |
| *agn43/bcsA/eaeA/fimC/fyuA/irp2/ompT/sitA* | 1 |
| *astA/bcsA/fimC/fyuA/irp2/iss/ompT/sitA* | 1 |
| *astA/bcsA/fimC/fyuA/irp2/ompT/sitA/tsh* | 4 |
| *agn43/astA/bcsA/fimC/fyuA/irp2/sitA* | 2 |
| *agn43/astA/bcsA/fimC/irp2/ompT/sitA* | 2 |
| *agn43/astA/bcsA/fimC/irp2/ompT/tsh* | 1 |
| *agn43/astA/bcsA/fyuA/irp2/ompT/tsh* | 1 |
| *agn43/astA/bcsA/fimC/iss/ompT/sitA* | 1 |
| *agn43/astA/bcsA/fimC/ompT/sitA/tsh* | 1 |
| *agn43/bcsA/eaeA/fimC/Ler/ompT/sitA* | 1 |
| *agn43/bcsA/fimC/fyuA/irp2/iss/sitA* | 2 |
| *agn43/bcsA/fimC/fyuA/irp2/ompT/sitA* | 4 |
| *agn43/bcsA/fimC/irp2/LT/ompT/sitA* | 1 |
| *bcsA/colv/fimC/hlyf/ompT/sitA/tsh* | 1 |
| *bcsA/colv/fyuA/irp2/iss/ompT/sitA* | 1 |
| *bcsA/fimC/fyuA/irp2/iss/ompT/sitA* | 2 |
| *agn43/astA/bcsA/fimC/hlyA/ompT* | 1 |
| *agn43/astA/bcsA/fimC/irp2/sitA* | 1 |
| *agn43/astA/bcsA/fimC/ompT/sitA* | 1 |
| *agn43/astA/bcsA/irp2/ompT/sitA* | 1 |
| *agn43/bcsA/eaeA/fimC/fyuA/ ompT* | 1 |
| *agn43/bcsA/fimC/fyuA/irp2/ompT* | 2 |
| *agn43/bcsA/fimC/fyuA/irp2/sitA* | 6 |
| *agn43/bcsA/fimC/fyuA/irp2/tsh* | 1 |
| *agn43/bcsA/fimC/irp2/iss/ompT* | 1 |
| *agn43/bcsA/fimC/irp2/ompT/sitA* | 2 |
| *agn43/bcsA/fimC/ompT/sitA/tsh* | 5 |
| *astA/bcsA/fimC/fyuA/irp2/tsh* | 1 |
| *astA/bcsA/fimC/ompT/sitA/tsh* | 1 |
| *bcsA/fimC/fyuA/irp2/ompT/sitA* | 4 |
| *agn43/astA/bcsA/fimC/fyuA* | 1 |
| *agn43/astA/bcsA/fimC/iss* | 1 |
| *agn43/astA/bcsA/fimC/ompT* | 5 |
| *agn43/astA/bcsA/iss/ompT* | 1 |
| *agn43/astA/bcsA/ompT/sitA* | 1 |
| *agn43/astA/irp2/sitA/tsh* | 1 |
| *agn43/bcsA//eaeA/fimC/ompT* | 2 |
| *agn43/bcsA/fimC/fyuA/irp2* | 5 |
| *agn43/bcsA/fimC/irp2/iss* | 1 |
| *agn43/bcsA/fimC/hlyA/ompT* | 1 |
| *agn43/bcsA/fimC/hlyf/ompT* | 1 |
| *agn43/bcsA/fimC/irp2/ompT* | 1 |
| *agn43/bcsA/fimC/iss/ompT* | 5 |
| *agn43/bcsA/fimC/fyuA/sitA* | 1 |
| *agn43/bcsA/fimC/irp2/sitA* | 3 |
| *agn43/bcsA/fimC/ompT/sitA* | 6 |
| *agn43/bcsA/fimC/iss/tsh* | 1 |
| *agn43/bcsA/fimC/ompT/tsh* | 1 |
| *agn43/bcsA/irp2/ompT/sitA* | 3 |
| *agn43/bcsA/ompT/sitA/tsh* | 1 |
| *astA/bcsA/fimC/irp2/sitA* | 1 |
| *bcsA/fimC/fyuA/irp2/ompT* | 2 |
| *bcsA/fimC/fyuA/irp2/sitA* | 2 |
| *bcsA/fimC/fyuA/ompT/sitA* | 2 |
| *bcsA/fimC/fyuA/stx2/tsh* | 1 |
| *bcsA/fimC/hlyf/iss/sitA* | 1 |
| *bcsA/fimC/irp2/ompT/sitA* | 4 |
| *bcsA/fimC/iss/ompT/sitA* | 2 |
| *bcsA/hlyf/iss/ompT/sitA* | 1 |
| *agn43/astA/bcsA/fimC* | 5 |
| *agn43/bcsA/fimC/fyuA* | 1 |
| *agn43/bcsA/fimC/iss* | 1 |
| *agn43/bcsA/fimC/ompT* | 11 |
| *agn43/bcsA/fimC/sitA* | 6 |
| *agn43/bcsA/ompT/sitA* | 1 |
| *agn43/bcsA/ompT/tsh* | 1 |
| *agn43/fimC/fyuA/irp2* | 1 |
| *astA/bcsA/fimC/irp2* | 1 |
| *astA/bcsA/fimC/iss* | 1 |
| *astA/bcsA/fimC/ompT* | 3 |
| *astA/bcsA/fimC/sitA* | 2 |
| *bcsA/fimC/fyuA/irp2* | 3 |
| *bcsA/fimC/fyuA/ompT* | 2 |
| *bcsA/fimC/irp2/ompT* | 2 |
| *bcsA/fimC/ompT/sitA* | 5 |
| *bcsA/fimC/stx2/tsh* | 1 |
| *agn43/bcsA/fimC* | 19 |
| *agn43/bcsA/ompT* | 2 |
| *agn43/bcsA/sitA* | 1 |
| *astA/bcsA/fimC* | 3 |
| *astA/bcsA/ompT* | 1 |
| *bcsA/fimC/fyuA* | 1 |
| *bcsA/fimC/irp2* | 1 |
| *bcsA/fimC/iss* | 1 |
| *bcsA/fimC/ompT* | 13 |
| *bcsA/fimC/sitA* | 1 |
| *bcsA/fimC/tsh* | 3 |
| *bcsA/irp2/ompT* | 1 |
| *agn43/bcsA* | 1 |
| *astA/bcsA* | 2 |
| *astA/ompT* | 1 |
| *bcsA/fimC* | 17 |
| *bcsA/sitA* | 2 |
| *bcsA* | 1 |

**
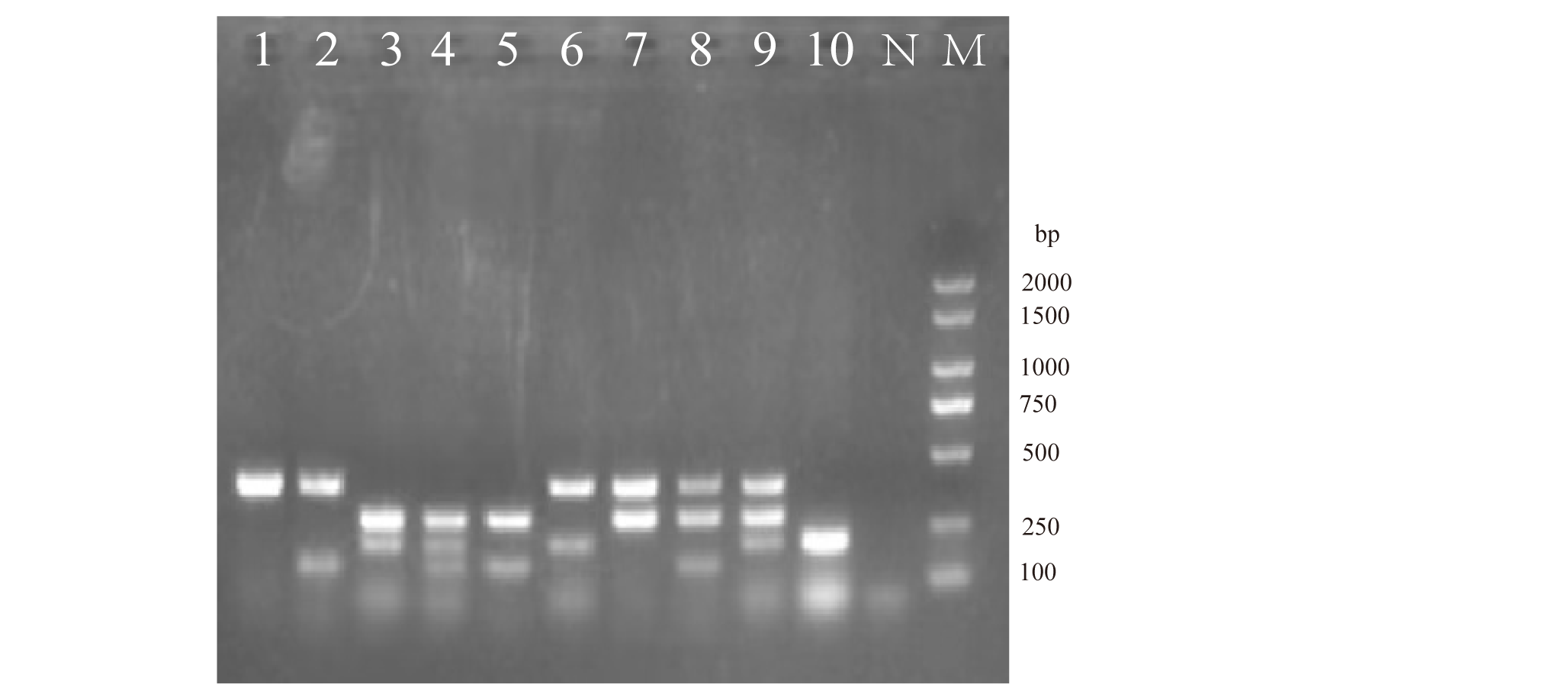
**

**Supplementary Figure 1.** **Quadruplex PCR profiles of** **phylogenetic typing.** Lane 1, Group A (+ - - -); Lane 2, Group B1(+ - - +); Lane 3, Group B2(- + + -); Lane 4, Group B2 (- + + +); Lane 5, Group B2(- + - +); Lane 6, Group A or group C(+ - + -); Lane 7, Group D or group E(+ + - -); Lane 8, Group D or group E(+ + - +); Lane 9, Group clade Ⅰ or group E(+ + + -); Lane 10, Group clade Ⅰ (- - + -); N, Negative control; M, DL 2000 DNA Marker. “+” indicates positively amplified, “-” indicates amplified.


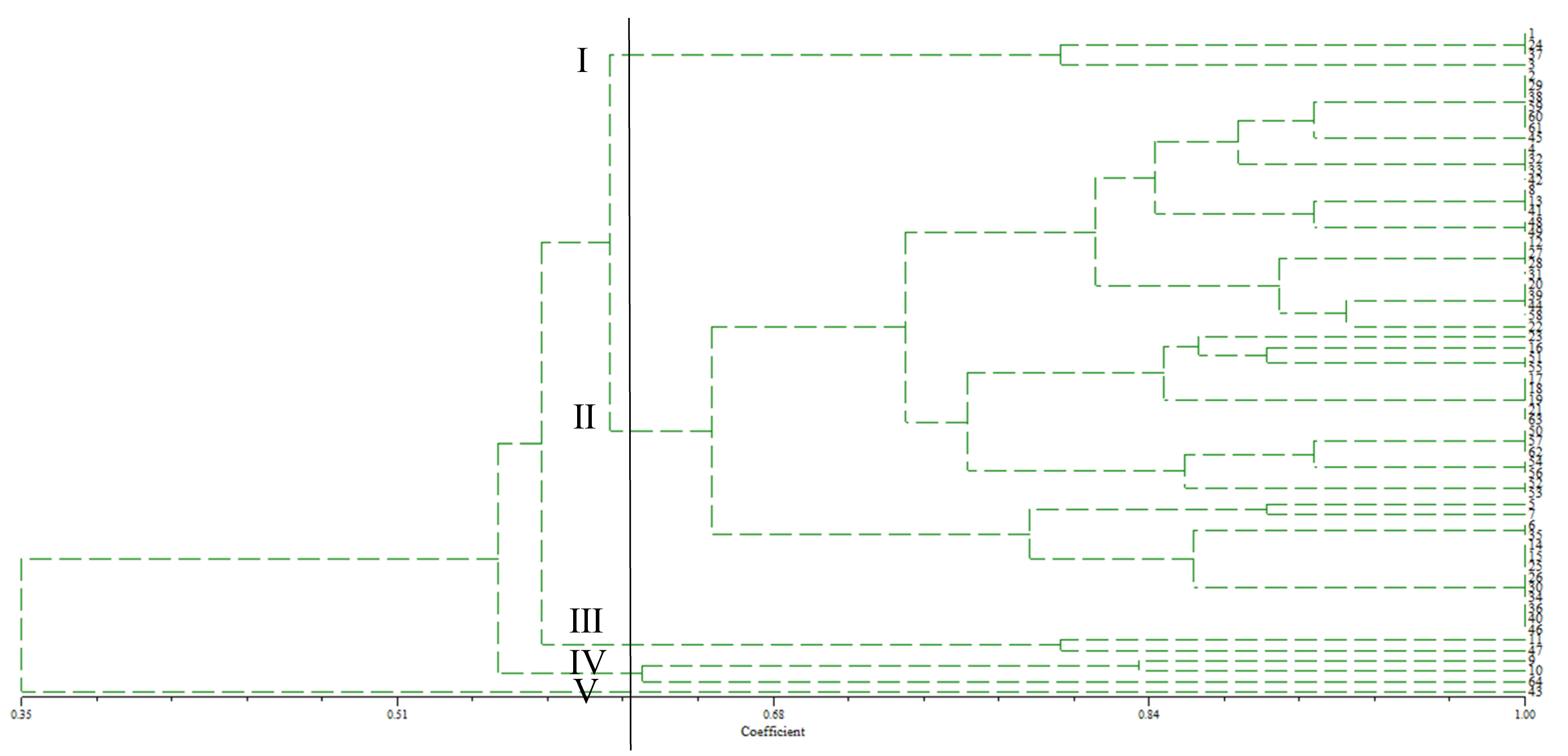


**Supplementary Figure 2** Cluster analysis dendrogram of diarrheagenic *E. coli*

**Reference**

Afsharnia, M., Naraghi, B., Mardaneh, J., Kianmehr, M., and Biglari, H. (2018). The data of *Escherichia coli* strains genes in different types of waste water. *Data Brief* 21, 763–766. doi: 10.1016/j.dib.2018.08.167

Akunda, I. K., Kariuki, D. W., Matulis, G., Mwaura, P., Maina, B., Mohammed, H., et al. (2023). Antimicrobial resistance patterns and characterisation of emerging beta-lactamase-producing *Escherichia coli* in camels sampled from Northern Kenya. *Vet Med Sci* 9, 1407–1416. doi: 10.1002/vms3.1090

Beeton, M. L., Chalker, V. J., Maxwell, N. C., Kotecha, S., and Spiller, O. B. (2009). Concurrent titration and determination of antibiotic resistance in ureaplasma species with identification of novel point mutations in genes associated with resistance. *Antimicrob Agents Chemother* 53, 2020–2027. doi: 10.1128/AAC.01349-08

Chen, S., Zhao, S., White, D. G., Schroeder, C. M., Lu, R., Yang, H., et al. (2004). Characterization of Multiple-Antimicrobial-Resistant *Salmonella* serovars isolated from retail meats. *Appl Environ Microbiol* 70, 1–7. doi: 10.1128/AEM.70.1.1-7.2004

Clermont, O., Lescat, M., O’Brien, C. L., Gordon, D. M., Tenaillon, O., and Denamur, E. (2008). Evidence for a human-specific *Escherichia coli* clone. *Environ Microbiol* 10, 1000–1006. doi: 10.1111/j.1462-2920.2007.01520.x

El-Badawy, M. F., Tawakol, W. M., El-Far, S. W., Maghrabi, I. A., Al-Ghamdi, S. A., Mansy, M. S., et al. (2017). Molecular identification of aminoglycoside-modifying enzymes and plasmid-mediated quinolone resistance genes among *Klebsiella pneumoniae* clinical isolates recovered from egyptian patients. *Int J Microbiol* 2017, 8050432. doi: 10.1155/2017/8050432

Gazal, L. E. S., Puño-Sarmiento, J. J., Medeiros, L. P., Cyoia, P. S., da Silveira, W. D., Kobayashi, R. K. T., et al. (2015). Presence of pathogenicity islands and virulence genes of extraintestinal pathogenic *Escherichia coli* (ExPEC) in isolates from avian organic fertilizer. *Poult Sci* 94, 3025–3033. doi: 10.3382/ps/pev278

Hu, L., Grim, C. J., Franco, A. A., Jarvis, K. G., Sathyamoorthy, V., Kothary, M. H., et al. (2015). Analysis of the cellulose synthase operon genes, *bcsA*, *bcsB*, and *bcsC* in Cronobacter species: Prevalence among species and their roles in biofilm formation and cell-cell aggregation. *Food Microbiol* 52, 97–105. doi: 10.1016/j.fm.2015.07.007

Jeong, Y.-W., Kim, T.-E., Kim, J.-H., and Kwon, H.-J. (2012). Pathotyping avian pathogenic *Escherichia coli* strains in Korea. *J Vet Sci* 13, 145–152. doi: 10.4142/jvs.2012.13.2.145

Johnson, J. R., and Stell, A. L. (2000). Extended virulence genotypes of *Escherichia coli* strains from patients with urosepsis in relation to phylogeny and host compromise. *J Infect Dis* 181, 261–272. doi: 10.1086/315217

Johnson, T. J., Wannemuehler, Y., Doetkott, C., Johnson, S. J., Rosenberger, S. C., and Nolan, L. K. (2008). Identification of minimal predictors of avian pathogenic *Escherichia coli* virulence for use as a rapid diagnostic tool. *J Clin Microbiol* 46, 3987–3996. doi: 10.1128/JCM.00816-08

Khorsi, K., Messai, Y., Hamidi, M., Ammari, H., and Bakour, R. (2015). High prevalence of multidrug-resistance in Acinetobacter baumannii and dissemination of carbapenemase-encoding genes blaOXA-23-like, blaOXA-24-like and blaNDM-1 in Algiers hospitals. *Asian Pac J Trop Med* 8, 438–446. doi: 10.1016/j.apjtm.2015.05.011

Kun Q. (2019) Detection of antimicrobial resistance and resistance genes of *E. coli* isolated from rabbits. [master's thesis]. Yangzhou province: Yangzhou University. (2019). (in Chinese)

Lescat, M., Clermont, O., Woerther, P. L., Glodt, J., Dion, S., Skurnik, D., et al. (2013). Commensal *Escherichia coli* strains in Guiana reveal a high genetic diversity with host-dependant population structure. *Environ Microbiol Rep* 5, 49–57. doi: 10.1111/j.1758-2229.2012.00374.x

Li X. (2019). The analysis on resistance characteristics of colistin resistance gene mcr-1-positive *Escherichia coli* from pigs. [master's thesis]. Henan province: Henan Agricultural University. (2019). (in Chinese)

Lin, T., Nomura, S., Someno, S., Abe, T., Nishiyama, M., Shiki, S., et al. (2023). Role of multidrug resistance and co-resistance on a high percentage of streptomycin resistance in *Escherichia coli* isolated from chicken meats in Japan. *J Vet Med Sci* 85, 832–836. doi: 10.1292/jvms.23-0135

Liu, G., Ding, L., Han, B., Piepers, S., Naqvi, S. A., Barkema, H. W., et al. (2018). Characteristics of *Escherichia coli* isolated from bovine mastitis exposed to subminimum inhibitory concentrations of cefalotin or ceftazidime. *BioMed Research International* 2018, 1–10. doi: 10.1155/2018/4301628

Ma, X., Guo, N., Ren, S., Wang, S., and Wang, Y. (2019). Response of antibiotic resistance to the co-existence of chloramphenicol and copper during bio-electrochemical treatment of antibiotic-containing wastewater. *Environ Int* 126, 127–133. doi: 10.1016/j.envint.2019.02.002

Mabika Mabika, R., Mounioko, F., Souza, A., and Yala, J. F. (2021). Phenotypic and molecular characterization of quinolone resistance of enteropathogens isolated from diarrhea in young children in Koula-Moutou/Gabon. *Egyptian Journal of Basic and Applied Sciences* 8, 345–353. doi: 10.1080/2314808X.2021.2001244

Manzoor, K., Rasool, F., Khan, N., Anjum, K. M., and Parveen, S. (2023). Resistance patterns of frequently applied antimicrobials and occurrence of antibiotic resistance genes in *Edwardsiella* tarda detected in edwardsiellosis-infected yilapia species of fish farms of punjab in Pakistan. *J Microbiol Biotechnol* 33, 668–679. doi: 10.4014/jmb.2301.01008

Mohamed, M. A., Shehata, M. A., and Rafeek, E. (2014). Virulence genes content and antimicrobial resistance in *Escherichia coli* from broiler chickens. *Vet Med Int* 2014, 195189. doi: 10.1155/2014/195189

O, C., Jk, C., E, D., and Dm, G. (2013). The Clermont *Escherichia coli* phylo-typing method revisited: improvement of specificity and detection of new phylo-groups. *Environmental microbiology reports* 5. doi: 10.1111/1758-2229.12019

Park, H., Kim, J., Ryu, S., and Jeon, B. (2022). The rate of frequent co-existence of plasmid-mediated quinolone resistance (PMQR) and extended-spectrum *β*-lactamase (ESBL) genes in *Escherichia coli* isolates from retail raw chicken in South Korea. *Food Sci Biotechnol* 31, 739–743. doi: 10.1007/s10068-022-01077-0

Ranjbar, R., Tabatabaee, A., Behzadi, P., and Kheiri, R. (2017). Enterobacterial repetitive intergenic consensus polymerase chain reaction (ERIC-PCR) genotyping of *Escherichia coli* strains isolated from different animal stool specimens. *Iran J Pathol* 12, 25–34. doi:10.30699/ijp.2017.21506

Rezanejad, M., Karimi, S., and Momtaz, H. (2019). Phenotypic and molecular characterization of antimicrobial resistance in Trueperella pyogenes strains isolated from bovine mastitis and metritis. *BMC Microbiol* 19, 305. doi: 10.1186/s12866-019-1630-4

Ruiz, J., Simon, K., Horcajada, J. P., Velasco, M., Barranco, M., Roig, G., et al. (2002). Differences in virulence factors among clinical isolates of *Escherichia coli* causing cystitis and pyelonephritis in women and prostatitis in men. *J Clin Microbiol* 40, 4445–4449. doi: 10.1128/JCM.40.12.4445-4449.2002

Sáenz, Y., Briñas, L., Domínguez, E., Ruiz, J., Zarazaga, M., Vila, J., et al. (2004). Mechanisms of resistance in multiple-antibiotic-resistant *Escherichia coli* strains of human, animal, and food origins. *Antimicrob Agents Chemother* 48, 3996–4001. doi: 10.1128/AAC.48.10.3996-4001.2004

Shivakumaraswamy, S. K., Deekshit, V. K., Vittal, R., Akhila, D. S., Mundanda, D. M., Mohan Raj, J. R., et al. (2019). Phenotypic & genotypic study of antimicrobial profile of bacteria isolates from environmental samples. *Indian J Med Res* 149, 232–239. doi: 10.4103/ijmr.IJMR_2097_17

Singh, T., Singh, P. K., Das, S., Wani, S., Jawed, A., and Dar, S. A. (2019). Transcriptome analysis of beta-lactamase genes in diarrheagenic *Escherichia coli*. *Sci Rep* 9, 3626. doi: 10.1038/s41598-019-40279-1

Wang Y., Tang C., Yu X., Wang Y., and Yu H. (2009). A multiplex PCR for detection of Shiga Toxin-producing *Escherichia coli* isolated from ducks. *Chinese Journal of Preventive Veterinary Medicine* 31, 780–784. (in Chinese)

Wilczyński, J., Stępień-Pyśniak, D., Wystalska, D., and Wernicki, A. (2022). Molecular and serological characteristics of avian pathogenic *Escherichia coli* Isolated from various clinical cases of poultry colibacillosis in Poland. *Animals* 12, 1090. doi: 10.3390/ani12091090

Wu L. (2018) Detection and analysis of drug resistance genes among *Escherichia coli* isolates from companion animals in Yangzhou area. [master's thesis]. Yangzhou province: Yangzhou University. (2018). (in Chinese)

Zhang, A., Wang, H., Tian, G., Zhang, Y., Yang, X., Xia, Q., et al. (2009). Phenotypic and genotypic characterisation of antimicrobial resistance in faecal bacteria from 30 Giant pandas. *Int J Antimicrob Agents* 33, 456-460.doi: 10.1016/j.ijantimicag.2008.10.030

Zhang, D., Zhang, Z., Huang, C., Gao, X., Wang, Z., Liu, Y., et al. (2018). The phylogenetic group, antimicrobial susceptibility, and virulence genes of *Escherichia coli* from clinical bovine mastitis. *J Dairy Sci* 101, 572–580. doi: 10.3168/jds.2017-13159

Zhong Q. (2013). The Detection of plasmid-mediated ESBLs and AmpC *β*-lactamase genes in *Escherichia coli* isolated from animals in Sichuan Province. [master's thesis]. Sichuan province: Sichuan Agricultural University. (2012). (in Chinese)
